# One-step synthesis of graphene containing topological defects

**DOI:** 10.1039/d5sc03699b

**Published:** 2025-09-09

**Authors:** Benedikt P. Klein, Matthew A. Stoodley, Joel Deyerling, Luke A. Rochford, Dylan B. Morgan, David Hopkinson, Sam Sullivan-Allsop, Henry Thake, Fulden Eratam, Lars Sattler, Sebastian M. Weber, Gerhard Hilt, Alexander Generalov, Alexei Preobrajenski, Thomas Liddy, Leon B. S. Williams, Mhairi A. Buchan, Graham A. Rance, Tien-Lin Lee, Alex Saywell, Roman Gorbachev, Sarah J. Haigh, Christopher S. Allen, Willi Auwärter, Reinhard J. Maurer, David A. Duncan

**Affiliations:** a Diamond Light Source, Harwell Science and Innovation Campus Didcot OX11 0DE UK david.duncan@nottingham.ac.uk; b Department of Chemistry, University of Warwick Gibbet Hill Road Coventry CV4 7AL UK r.maurer@warwick.ac.uk; c Physics Department E20, TUM School of Natural Sciences, Technical University of Munich James-Franck-Straße 1 85748 Garching Germany; d Department of Earth Sciences, University of Cambridge Downing Street Cambridge CB2 3EQ UK; e National Graphene Institute, University of Manchester Oxford Road Manchester M13 9PL UK; f Institute of Chemistry, Carl von Ossietzky University Oldenburg Carl-von-Ossietzky-Straße 9-11 26111 Oldenburg Germany; g MAX IV Laboratory, University of Lund Fotongatan 2 224 84 Lund Sweden; h School of Chemistry, University of Nottingham, University Park Nottingham NG7 2RD UK; i School of Chemistry, University of Glasgow, University Avenue Glasgow G12 8QQ UK; j School of Physics & Astronomy, University of Glasgow, University Avenue Glasgow G12 8QQ UK; k School of Chemistry, University of St. Andrews N. Haugh, St Andrews KY16 9ST UK; l Nanoscale & Microscale Research Centre, University of Nottingham, University Park Nottingham NG7 2RD UK; m School of Physics & Astronomy, University of Nottingham, University Park Nottingham NG7 2RD UK; n Department of Materials, University of Oxford Parks Road Oxford OX1 3PH UK; o Department of Physics, University of Warwick Gibbet Hill Road Coventry CV4 7AL UK

## Abstract

Chemical vapour deposition enables large-domain growth of ideal graphene, yet many applications of graphene require the controlled inclusion of specific defects. We present a one-step chemical vapour deposition procedure aimed at retaining the precursor topology when incorporated into the grown carbonaceous film. When azupyrene, the molecular analogue of the Stone–Wales defect in graphene, is used as a precursor, carbonaceous monolayers with a range of morphologies are produced as a function of the copper substrate growth temperature. The higher the substrate temperature during deposition, the closer the resulting monolayer is to ideal graphene. Analysis, with a set of complementary materials characterisation techniques, reveals morphological changes closely correlated with changes in the atomic adsorption heights, network topology, and concentration of 5-/7-membered carbon rings. The engineered defective carbon monolayers can be transferred to different substrates, potentially enabling applications in nanoelectronics, sensorics, and catalysis.

## Abstract 2

Temperature-controlled CVD growth with an azupyrene precursor enables tunable defect concentration in graphene-like films, with high defect homogeneity.

## Graphical abstract

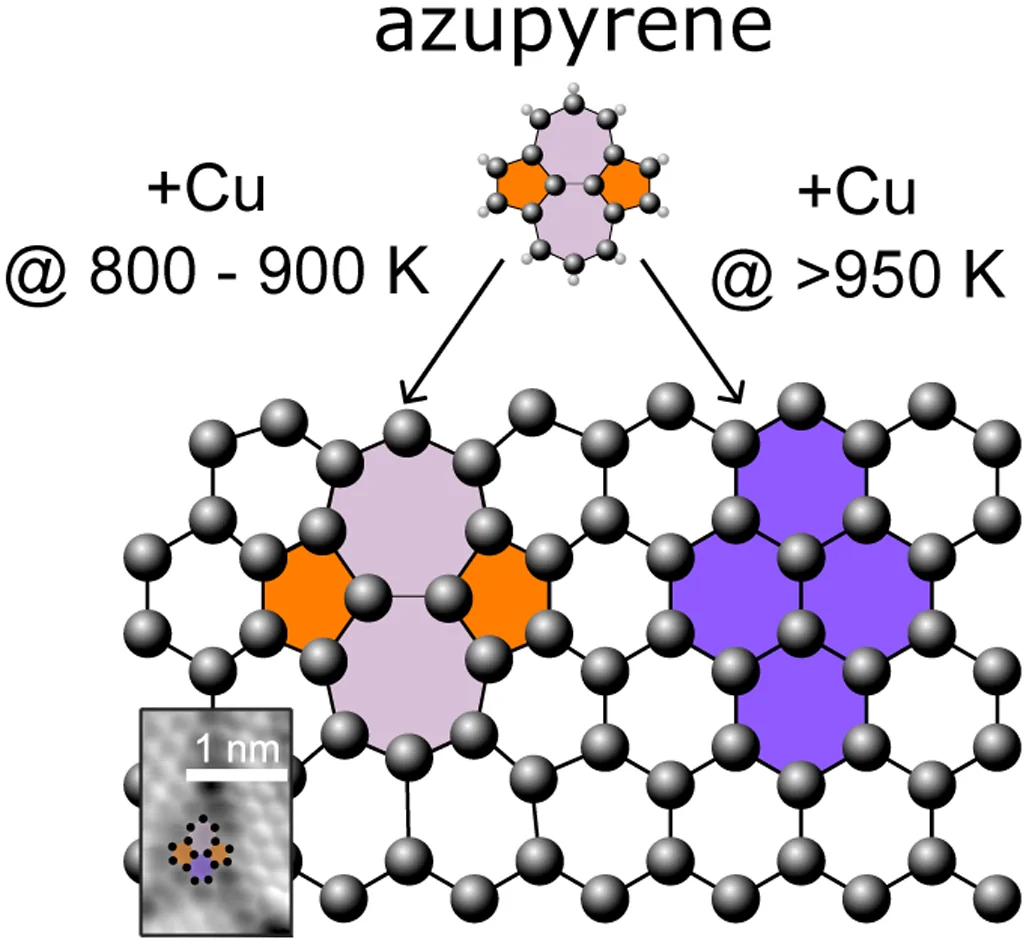

Chemical vapour deposition (CVD) procedures have realised single-domain growth of ideal graphene on the meter scale.^1^ However, for many applications, notably as a catalytic support material,^2^ a battery electrode,^3^ a gas sensor^4,5^ or an electronic component,^6^ perfectly crystalline graphene is not an ideal material. The inclusion of defects into graphene is often necessary to improve specificity of binding to graphene, for catalytic or gas sensor applications, or to modify graphene's electronic and magnetic properties for nanoelectronics or valleytronics applications.^2–6^ For example, a Stone–Wales defect, which replaces four 6-membered rings with two 5- and two 7-membered rings, increases the strength of interaction between graphene and its substrate;^7,8^ vacancy and heteroatom defects in graphene can introduce magnetic order at finite temperature.^9^

Current methods for including defects in graphene are, generally, post-processing modifications of ideal graphene or graphene oxide,^5,10–12^ though these methods lack control and have poor defect homogenity.^13^ Ullman coupling methods^14^ can yield highly crystalline films, in particular for covalent organic frameworks. However, Ullman coupling requires halide groups that typically contaminate the product^15–17^ and can have a deleterious effect on the film quality at elevated temperatures.^18^ Contamination and defect inhomogeneity will hinder studies into elucidating the material properties induced by the defects and, for gas sensor and catalytic support applications, spoil their specificity. Thus, better controlled inclusion of defects is necessary to create reproducible defective graphene films.

One-step growth methods based on CVD, over a copper substrate, are the most common approach to growing ideal graphene^19–21^ and recent work by Amontree *et al.*^22^ reinforced the importance of performing graphene CVD growth in an oxygen-free environment, such as ultra-high vacuum (UHV), to obtain high quality and reproducible film growth. While few CVD studies have focused on deliberate inclusion of defects,^23–25^ the choice of precursor is known to have a significant effect on the growth temperature needed to create ideal graphene,^1,26–28^ thus precursor modification is a promising route for deliberate inclusion of defects. Azupyrene (Fig. 1c, inset) can be considered a molecular analogue of the Stone–Wales defect and, were its topology retained in the CVD process, topological defects would be induced into the resulting graphene-like film.

**Fig. 1:**
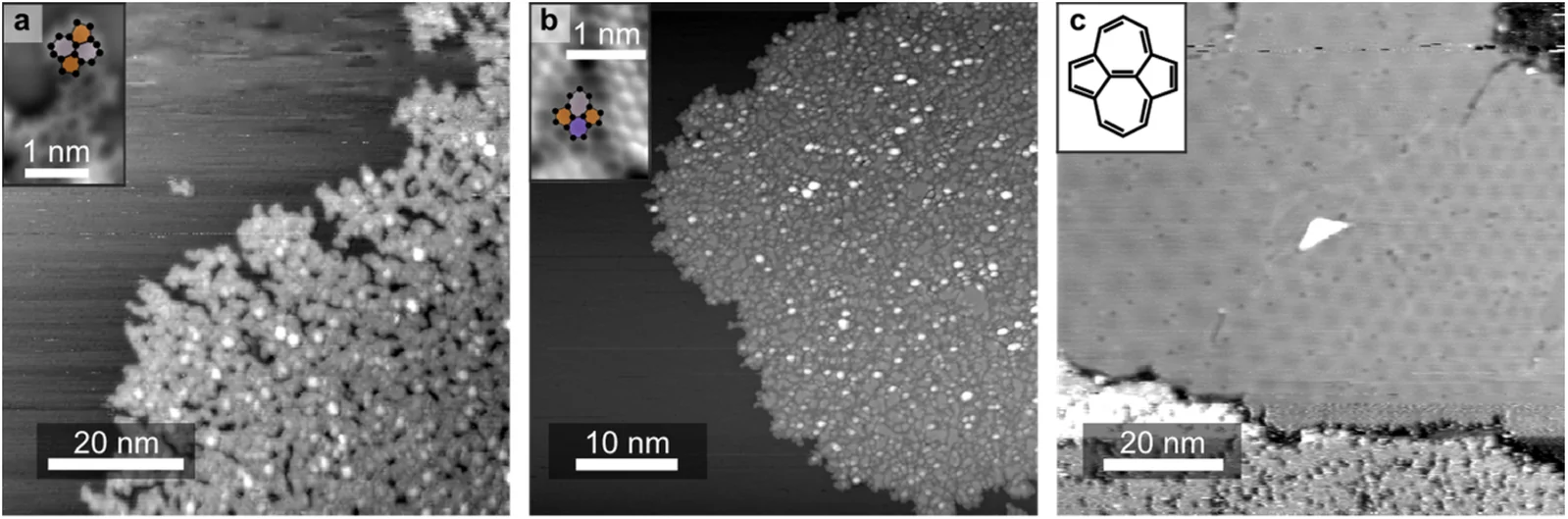
Microscopy measurements of the morphological differences in the carbonaceous layers as a function of the growth temperature. STM measurements of (a) a dendritic film, (b) a defective film and (c) ideal graphene. Insets in (a) and (b) show atomically resolved nc-AFM measurements of (a) a dendritic and (b) a defective film, highlighting 5-membered (dark orange) rings, and 7-membered (light purple) rings and 6-membered (dark purple) rings. The inset in (c) shows the molecular structure of azupyrene. [STM parameters: (a) *U*_tip_ = +1.5 V, *I*_tunnel_ = 1.25 nA; (b) *U*_tip_ = −1.9 V, *I*_tunnel_ = 0.5 nA; (c) *U*_tip_ = −1.5 V, *I*_tunnel_ = 0.8 nA]. Further STM measurements of the samples shown in panel (a and b), taken over a larger area of the surface, indicating the lack of any bi-layer formation, are shown in Fig. S2 in the SI.

We demonstrate that CVD growth on copper substrates with an azupyrene precursor indeed results in high concentrations of 5-/7-defect sites in a graphene-like film. The defect type is present with a high homogeneity, with vanishingly few alternate defect species present. We obtain continuous monolayer films that can be successfully transferred from their growth substrate onto other supports. These films are obtained without contamination by heteroatoms and at comparatively mild substrate temperatures (∼900 K).

## Experiment

The defective graphene samples were grown *in situ* under UHV conditions in the low 10^−10^ mbar pressure range. Four chambers were utilised for the sample growth. The growth chambers on the permanent end-station of beam line I09 at Diamond Light Source, UK, the growth chamber on the permanent end-station of the FlexPES beamline at MAX IV, Sweden, the growth chamber on the “surface interface laboratory” at Diamond Light Source, and the growth chamber attached to the non-contact atomic force microscopy (nc-AFM) system at the Technical University of Munich. These chambers are standard UHV growth vessels, containing facilities for sputtering and annealing samples in ultra-high vacuum. The Cu(111) surface was prepared by sputtering (*V* = 1 keV, *p* = 1–5 × 10^−5^ mbar Ar) and annealing (*T* = 1000 K). The cleanliness of the crystal was assessed by X-ray photoelectron spectroscopy (XPS), low energy electron diffraction (LEED), and scanning tunnelling microscopy (STM) depending on the system. During growth, the Cu(111) substrate was held at different temperatures and exposed to a molecular flux of the precursor. The precursor azupyrene (synthesis procedure see ref. 29) was degassed thoroughly under high vacuum to remove volatile impurities prior to the experiments. It was deposited *via* a home-built line-of-sight capillary doser (described in ref. 27, a rough schematic showing the growth geometry is shown in Fig. S1 in the Supplementary Information, SI), with the end of the capillary positioned as close to the sample surface as possible under the chamber geometry (within a few millimetres). During deposition, azupyrene was held at a temperature of 25 to 60 °C depending on the deposition geometry and to enable comparable deposition time and coverage. Further experimental and computational details can be found in the SI, §1.

## Results and discussion

### CVD growth of defective carbon networks

Carbonaceous networks were synthesised by CVD of the aromatic precursor azupyrene onto a hot Cu(111) surface. At room temperature, azupyrene adsorbs molecularly on the surface.^8^ The adsorption structure and electronic properties of the molecular overlayer have previously been discussed in ref. 8 and 30. At highly elevated substrate temperatures, ∼1000 K, as previously shown,^27,31^ azupyrene forms ideal graphene that demonstrate moiré superstructures, as observed by scanning tunnelling microscopy (STM, Fig. 1c). Formation of this film must involve surface-catalysed dehydrogenation and Stone–Wales rearrangement of the 5-/7-membered rings to form a network of 6-membered rings.

At intermediate substrate temperatures, dehydrogenative coupling of azupyrene to form on-surface networks is observed. The film morphology, measured by STM, (Fig. 1) varies as a function of growth temperature. Between 700 and 850 K, a dendritic network is formed, imaged (Fig. 1a) as bright finger-like protrusions separated by dark regions of the underlying substrate. Bond-resolved, CO-functionalised^32^ non-contact atomic force microscopy (nc-AFM) measurements (Fig. 1a, inset) show that the 5-/7-topology of azupyrene is retained in these dendrites. Pentagons and heptagons will not tessellate on a plane, thus without significant Stone–Wales re-arrangement an open dendritic network would be expected.

At higher temperatures (∼850–950 K) highly corrugated, closed two-dimensional islands are formed (Fig. 1b). To tessellate closed films, a large proportion of 6-membered rings must form on the surface and, indeed, the nc-AFM measurements (Fig. 1b, inset) show 5-/7-membered ring defects embedded into a lattice of 6-membered rings. Herein, these closed films are referred to as defective films.

Deposition at elevated substrate temperatures proved to be crucial for obtaining high coverage, closed films. Prior work has shown that only ∼50% of molecular azupyrene thermally desorbs from the surface,^8^ thus it may be expected that post annealing of molecular azupyrene on Cu would result in comparable quality films to that shown in Fig. 1, if at a somewhat lower coverage. However, depositing a saturated molecular monolayer at room temperature and post annealing to ∼725–820 K resulted in wire-like growth (SI, Fig. S3). These wires are roughly 1.5–2.5 nm in width, but can extend for tens of nanometres in length. Ignoring areas where different wires seem to have overlapped during growth, there is no indication of 2D-like growth.

### Local defect topology in networks

**Fig. 2:**
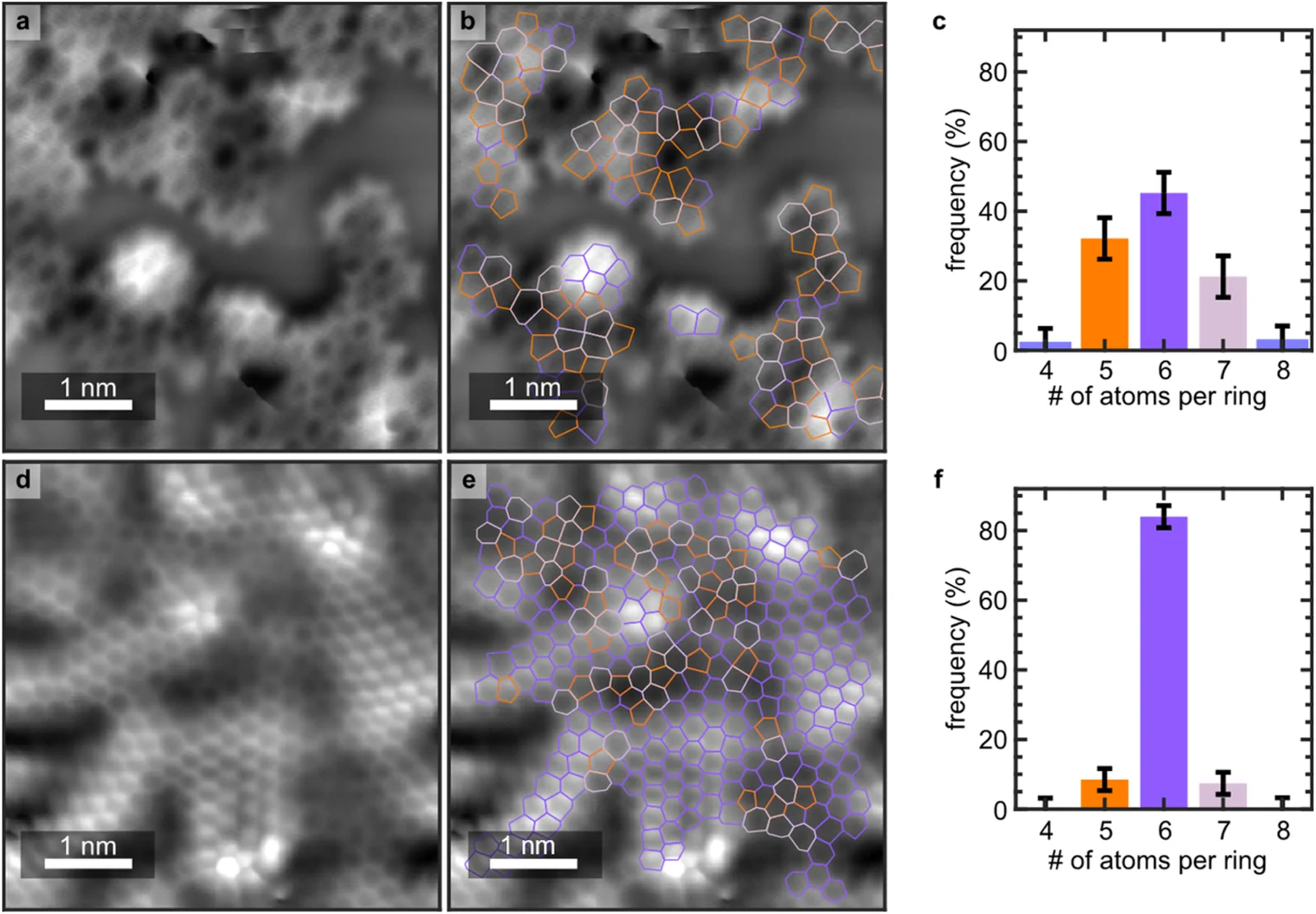
Large scale non-contact atomic force microscopy (nc-AFM) demonstrating the defect homogeneity of the topological defects in dendritic and defective films. nc-AFM measurements of (a and b) the dendritic film and the (d and e) defective film. Overlayed in panels (b) and (e) are the C–C bonds, 5-membered rings are dark orange, 7-membered are light purple and 6-membered are dark purple. Histograms show the relative frequency of 4- to 8-membered rings for (c) dendritic and (f) defective films. Imaging conditions for the nc-AFM measurements are in the Methods. Note: the color scale of panel (d) is set to accentuate the topology in the lower areas of the region, saturating the measurement in the highest region, this panel is reproduced in Fig. S11 of the SI without such rescaling.

The theoretical maximum concentration of 5-/7-defects in a closed two-dimensional film is the phagraphene film,^33,34^ where 40% of all rings are 5- or 7-membered. Large area nc-AFM measurements for a dendritic (Fig. 2a–c) and a defective film (Fig. 2d–f) indicate that over 50% and 15%, respectively, of all rings are 5-/7-membered. [NB. linking two azupyrene motifs can result in either a 6- or 5-membered ring (SI, §2)]. While different types of 5/7 defects are observed (*i.e.* other than Stone–Wales), defects with a differing topology (*e.g.* 4- or 8-membered) are vanishingly rare (<1% of all observed rings) in both films. Enumerating the 4- to 8-membered rings in a dendritic film measured over 59.6 nm^2^ (see Fig. 2a–c and S7 in the SI) yields a distribution of 46 ± 4% 6-membered rings, 32 ± 6% 5-membered rings, 21 ± 6% 7-membered rings, and 1 ± 6% 4- or 8-membered rings (total sample size, *N*, 283). Similarly enumerating the defective film over 107.5 nm^2^ (*N* = 999, see Fig. 2d–f and S8–S10 in the SI) yields a distribution of 84 ± 3% 6-membered rings, 9 ± 3% 5-membered rings, and 7 ± 3% 7-membered rings. No vacancy defects were observed. These values are summarised in Table S2 in the SI. The nc-AFM images are inherently local in scale, but they indicate a clear trend – that the dendritic films have a higher relative coverage of 5- and 7-membered rings than the defective films. These data also unequivocally indicate that 5- and 7-membered rings are still present in the closed islands identified as the defective films and, while the local nature of this measurement cannot exclude that there are 4- or 8-membered rings elsewhere in the prepared film, the relatively large number of counted rings suggest the presence of any such rings is statistically insignificant.

To form defective closed networks grown from azupyrene rather than dendritic networks requires Stone–Wales re-arrangement. The observation of dendritic networks at lower growth temperatures is consistent with the assumption that the energetic barrier for such re-arrangement is higher than the energy required to dehydrogenatively couple molecules. Density functional theory (DFT) calculations performed for gas phase reactions of azupyrene molecules indeed confirm that the barrier for re-arrangement is substantially higher than the energy required to couple two azupyrene molecules *via* sequential hydrogen atom elimination (see SI, §2). These calculations suggest that, at high enough growth temperatures, healing of Stone–Wales defects will proceed alongside the growth of the film. When the rate of defect annealing surpasses the rate of formation of new defects as part of growth processes, the concentration of 5-/7-rings is reduced and eventually a closed film can form.

### Spectroscopic signal of defective films

**Fig. 3:**
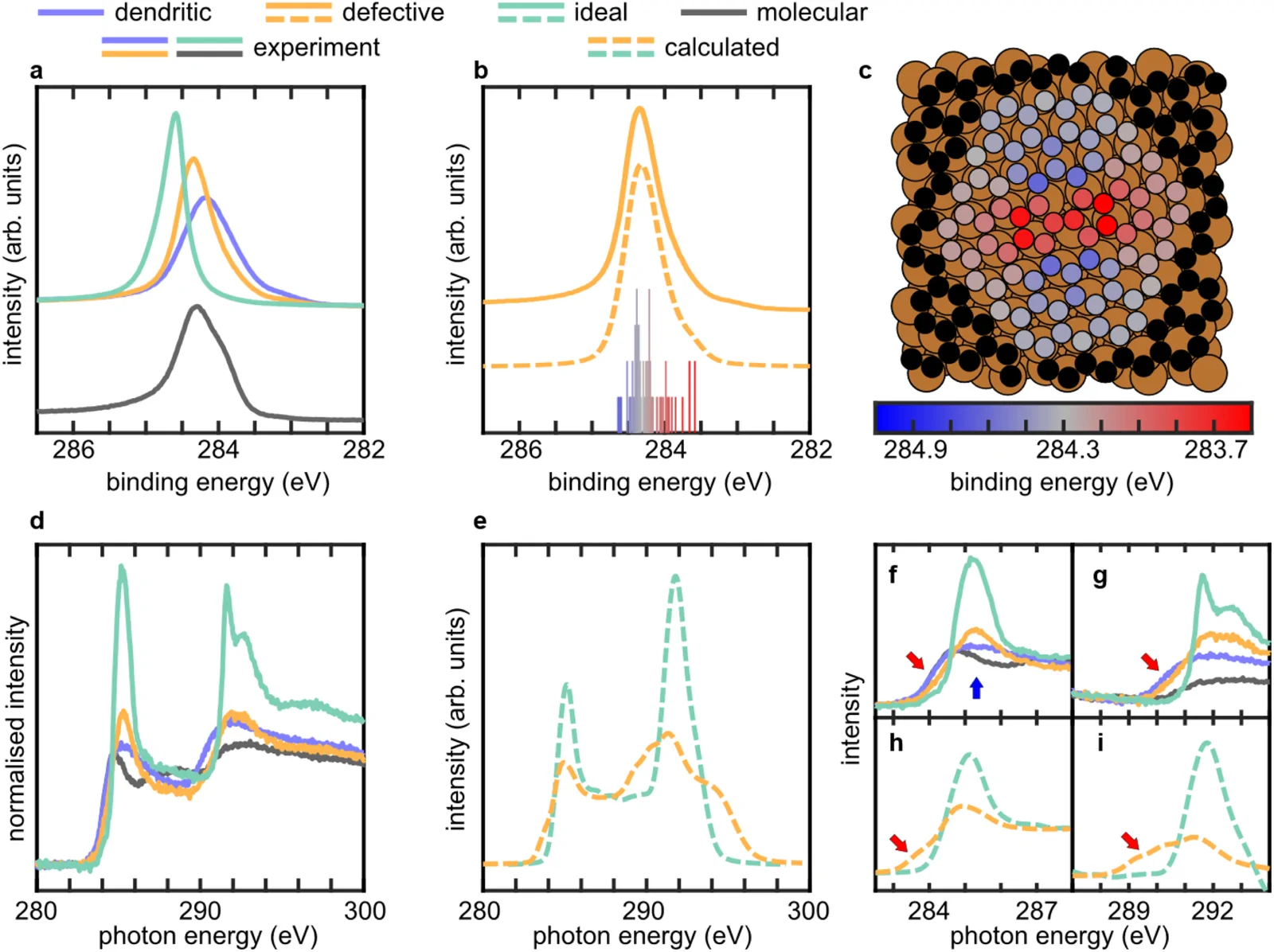
Spectroscopic data for graphene-like networks grown on Cu(111) compared to DFT simulation results. (a) Experimental SXPS data, (b) comparison of experimental and calculated XPS for defective graphene, with a histogram of the calculated binding energies, without broadening, using the same color scale as shown in panel (c) and (c) top view depiction of the structure employed in the DFT calculations, showing the binding energy of each calculated atom as per the indicated color scale. (d) Experimental and (e) DFT-simulated C K-edge NEXAFS spectra. Zoomed-in panels of the (f and h) π* and (g and i) σ* regions for the (f and g) experimental and (h and i) calculated C K-edge NEXAFS spectra. Further details are in SI, §9. Arrows indicate the regions of the spectra discussed in the main text.

Spectroscopic measurements, supported by simulated spectra, show signatures associated with the presence of 5-/7-defects. Soft X-ray photoelectron spectroscopy (SXPS, Fig. 3a) indicates a correlation between the binding energy (BE) of the primary peak in the C 1s SXP spectra and the growth temperature and, thus, the concentration of 6-membered rings in the CVD-grown film. The C 1s SXP spectra of molecular azupyrene and graphene adsorbed on Cu(111) (Fig. 3a) are consistent with reports in the literature^8,27,30,35^ (further discussion in the SI, §3). The C 1s SXP spectrum of the dendritic phase is broader than that of the molecular phase, with a small shoulder at lower BE. The whole spectrum has a BE lower than both graphene and azupyrene. On the high BE tail, the spectrum has a noticeable asymmetry, similar to, though slightly smaller than, that of molecular azupyrene. This asymmetry indicates that an electronic hybridisation with the substrate is, to some degree, present in the dendritic films (see SI, §2). The C 1s SXP spectrum of the defective film has a BE between that of the dendritic phase and ideal graphene. The main peak is narrower than both the dendritic and molecular phase spectra. The shoulder at lower BEs is still present but is weaker compared to the dendritic phase. The asymmetry in the high BE tail is not present for the defective films, which may indicate an electronic decoupling of the defective film from the substrate compared to the dendritic phase. Excluding a small transient Sn or Sb contamination, no heteroatoms were observed in the SXPS. SXPS was also used to demonstrate that the growth of the film is self-limited to a monolayer at high coverage (∼40C atoms per nm^2^), see SI, §4. This self-limitation will be driven by the film inhibiting the access of the precursor to the substrate that is catalysing the formation of these films.

The C K-edge NEXAFS spectra of molecular azupyrene and graphene (Fig. 3d) are in agreement with literature data (further discussion in the SI, §5).^8^ The C K-edge NEXAFS of the dendritic phase (Fig. 3d, f and g) are similar to that of molecular azupyrene, but with broadened features throughout and, in the π* region, an increased absorption rate at the photon energy of the graphene π* peak (blue arrow, Fig. 3f). In the σ* region of the spectrum, the dendritic phase has a feature at a lower photon energy than observed for either the molecular phase or ideal graphene (red arrow, Fig. 3g). The spectrum of the defective phase is more comparable to graphene, specifically π* features are more pronounced (blue arrow, Fig. 3f). While the onset of the π* region is, like the molecular and dendritic phase, at a lower photon energy than ideal graphene (red arrow, Fig. 3f), the intensity of this feature is reduced. In the σ* region, the defective film has a sharp feature similar to graphene, but the lower photon energy feature (red arrow, Fig. 3g), that was observed in the dendritic phase, is also present.

Simulated XPS data (DFT calculations, computational details in SI §6 and §7) of a Stone–Wales defect embedded in a graphene lattice qualitatively agree well with the experimentally measured defective SXPS (Fig. 3b). These simulations show that the XPS signals attributed to the defect atoms exhibit strong positive and negative BE shifts (Fig. 3b and c), with increasingly smaller shifts for atoms that are further from the defect. The 5-membered ring C atoms are shifted to lower BEs, while 7-membered ring C atoms are weakly shifted to higher BEs, interpreted as the rings being electron rich or poor, respectively.^7^ The BE shift of the 7-membered ring is larger in a free-standing Stone–Wales defect (Fig. S17b in SI).

Simulated NEXAFS data (DFT calculations, computational details in SI, §6 and §8) of carbon atoms within the Stone–Wales defect, compared against those within ideal graphene, on Cu(111), also show good qualitative agreement with experiment (Fig. 3d–i). As in the experimental data, these simulated spectra indicate that the presence of the Stone–Wales defect results in a broadening of the spectral features, as well as the introduction of features at lower photon energies than ideal graphene. These effects are observed in both the π* and σ* regions (red arrows, Fig. 3h and i, respectively), but, in particular, the feature at lower photon energies in the σ* region is only found experimentally for the dendritic and defective phases (red arrow, Fig. 3g). This feature is predicted by the theoretical calculations to be associated with the 5-/7-membered rings in the Stone Wales defect and may be a spectral fingerprint for 5-/7-defects.

### Quantitative structure of carbon films

The vertical adsorption structure of the grown films was probed by normal incidence X-ray standing waves (NIXSW, details in SI, §10),^36^ Analysis of NIXSW yields two structural parameters, the coherent fraction (*f*_H_) and the coherent position (*p*_H_). Broadly, *f*_H_ is an order parameter, *f*_H_>∼0.7 indicates a single adsorption height, *f*_H_<∼0.7 indicates multiple adsorption heights.^37^ The *p*_H_ is related to the mean adsorption height (*h*_H_) by eqn (1) in SI, §10.

**Fig. 4:**
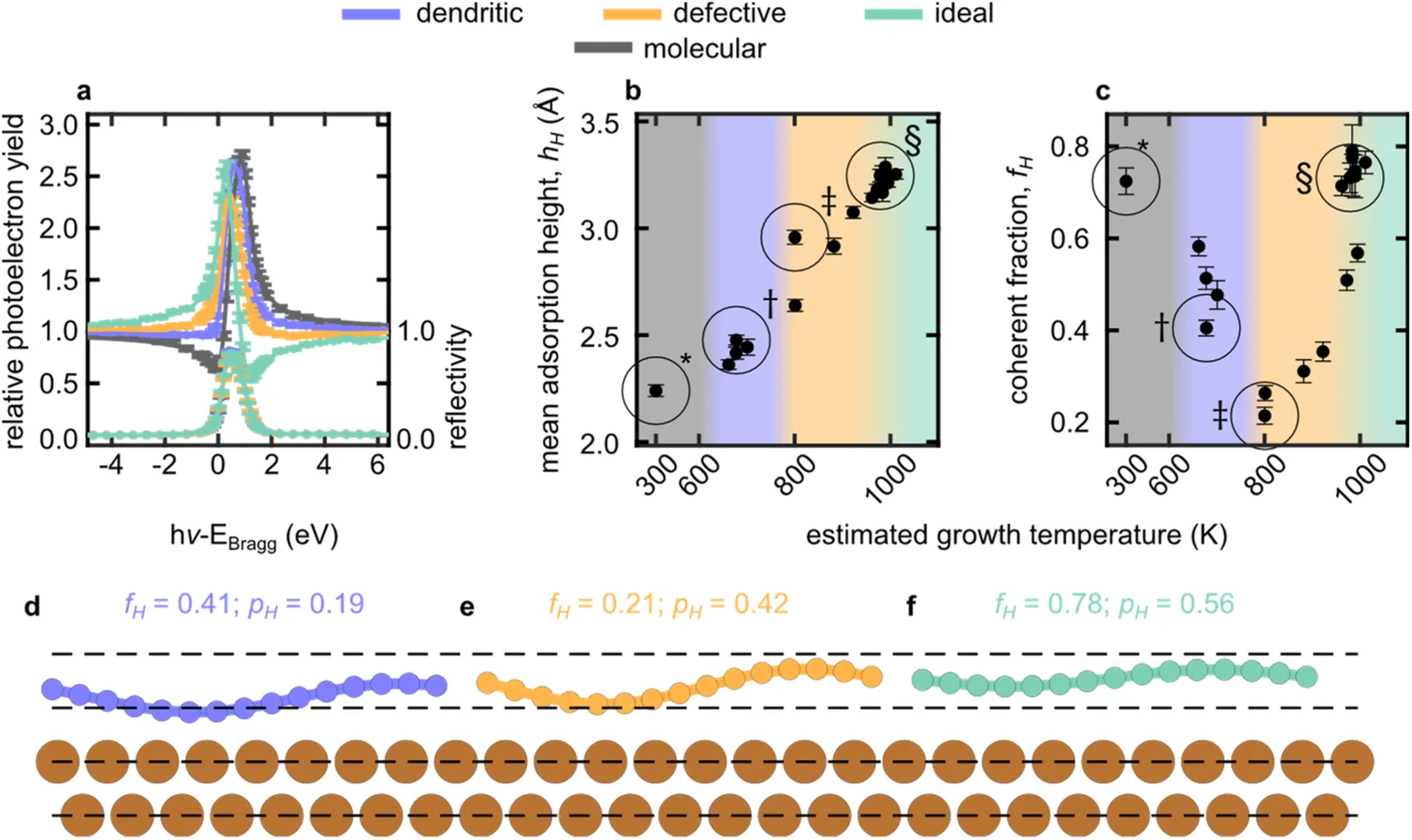
Structural data for graphene-like networks grown on Cu(111) at varying temperature. (a) Exemplary NIXSW yield curves, dependence of (b) mean adsorption height (*h*_H_) and (c) coherent fraction (*f*_H_) on the growth temperature. Data marked in panels (b and c) with * (molecular), † (dendritic), ‡ (defective) or § (graphene) correspond, respectively, to the NIXSW data displayed in (a) and were acquired from the same samples as the SXPS data shown in Fig. 3a and STM data in Fig. 1a–c. The temperature calibration is described in SI, §11. The SXPS and NIXSW data corresponding to panels b and c are shown in Fig. S19–S25 in the SI. Also shown is an illustration of the measured *f*_H_ and *p*_H_ for the (d) dendritic, (e) defective and (f) graphene phases with a simple sinusoidal. The *d*_111_ spacing is indicated with dashed lines.

The NIXSW of graphene and azupyrene adsorbed on Cu(111) are discussed in detail in prior work,^30,31^ but are compared here with respect to exemplar NIXSW data for the dendritic and defective phases (Fig. 4a) measured from the same samples as the STM images in Fig. 1. The NIXSW data show clear and stark differences, related to the different structures present on the surface in each phase.

The resulting mean adsorption heights and coherent fractions of nineteen different samples are shown in Fig. 4b and c. Excluding molecular azupyrene, *h*_H_ increases roughly linearly with growth temperature. The lowest (highest) adsorption heights, corresponding to the lowest (highest) growth temperatures, are similar to that of molecular azupyrene (ideal graphene, respectively). This variation suggests that the interaction between the substrate and the film decreases as the relative coverage of 5-/7-membered rings decreases. For all defective and dendritic phases, *f*_H_ is low (*f*_H_ = 0.2–0.6), with its minimum near the dendritic to defective transition, indicating multiple adsorption heights.^37^ Notably, the adsorption height of graphene and azupyrene differ by approximately half of the (111) layer spacing of copper (2.0871 Å). As discussed in SI, §10, an equal mixture of such adsorption heights would result in a *f*_H_ of 0. Thus, roughly, the variation in *f*_H_ with increasing growth temperature can be understood as the fraction of atoms at an *h*_H_ of molecular azupyrene decreasing in favour of those at the *h*_H_ of graphene. Illustrating the measured *p*_H_ and *f*_H_ of the dendritic, defective and ideal graphene films with a simple sinusoidal curve, yields the corrugations displayed in Fig. 4d–f.

Our results are thus consistent with the idea put forth in our prior work^8^ that Stone–Wales defects interact with metallic species more strongly than ideal graphene alone. Specifically, the correlation between adsorption height, measured by NIXSW, and the relative defect concentration, obtained from our nc-AFM measurements, indicate that the greater the concentration of the 5-/7-defect sites in the grown layer, the more strongly the film is bound to the surface. Conversely, the more graphene-like the film is (*i.e.* as the number of 5-/7-defects decreases), the more weakly the film as a whole interacts with the underlying substrate.

### Generating freestanding defective films

**Fig. 5:**
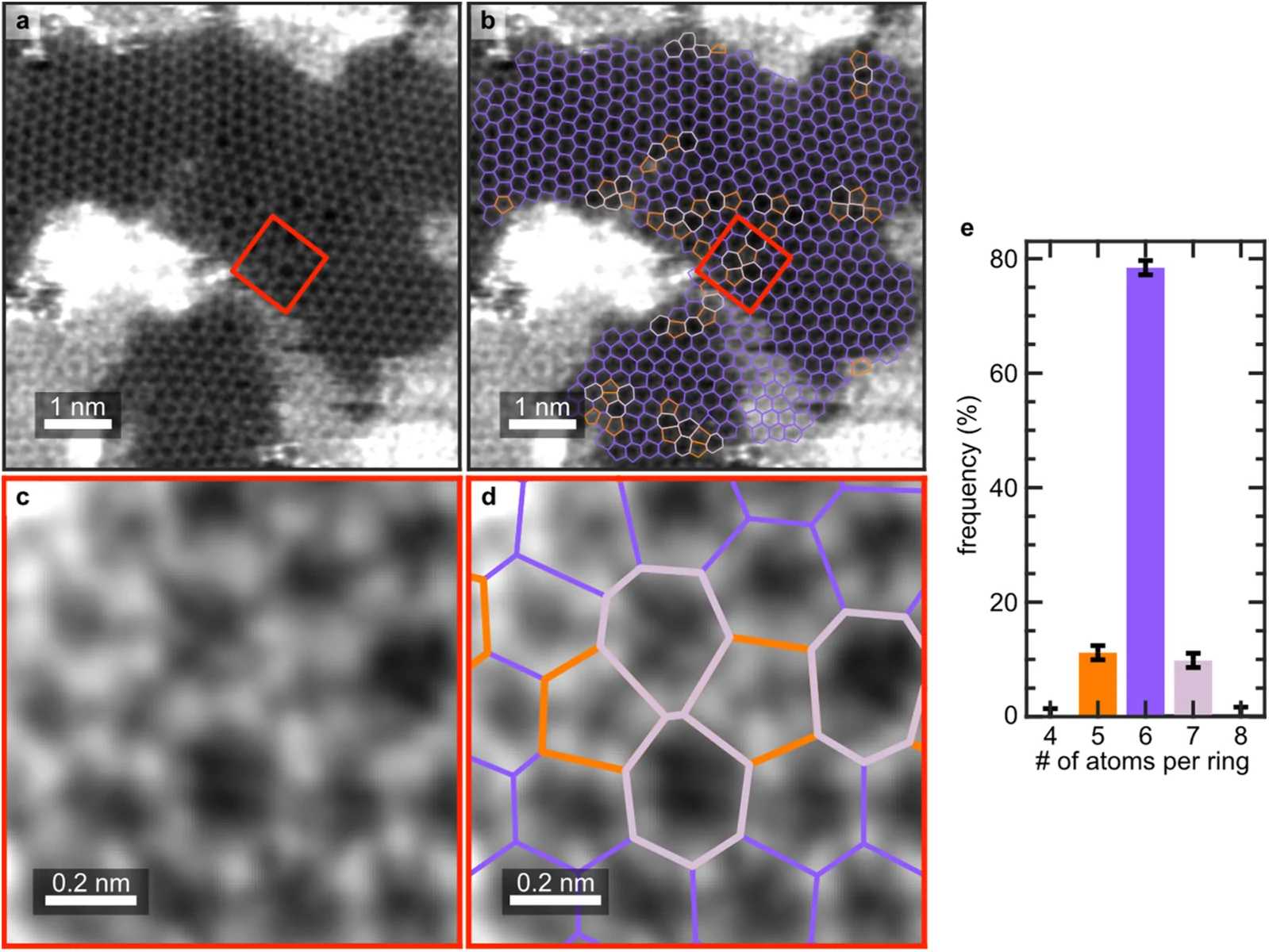
Annular dark-field scanning transmission electron microscopy (ADF-STEM) showing the retention of 5-/7-topological defects after transfer onto a TEM grid. (a–d) Atomically resolved ADF-STEM measurements of a defective film. Overlayed in panels (c) and (d) are the C–C bonds, following the same color legend as Fig. 2. A single Stone–Wales defect is highlighted in panels (b) and (d) and corresponds to the red box indicated in (a and c). (e) Histogram showing the relative frequency of 4- to 8-membered rings.

For many applications of defective graphene-like films, the presence of a copper growth substrate is deleterious and the use of single crystal growth substrates is undesired. Thus, as a proof of concept a defective film was grown on copper foil and transferred onto a silicon nitride TEM grid (based on ref. 38). As with the samples grown on the Cu(111) single crystal, the samples grown at high coverage on the Cu foil nominally covered the entire surface of the foil, without any indication of bilayer formation, as shown by scanning electron microscopy (SEM), see Fig. S26 in the SI. At all locations that the Cu growth substrate was etched away for transfer onto TEM grids, continuous films were observed, see Fig. S27 in the SI. In the etched areas that, themselves, were roughly 1 mm in scale the entire visible area was dominated by closed films, indicating that millimetre scale growth required for device-based studies was achieved. Annular dark-field scanning transmission electron microscopy (ADF-STEM) measurements over 1406 nm^2^ (*N* = 6505, Fig. 5 and S26–30 in the SI) demonstrate that the 5-/7-membered topological defects are present (11 ± 1% and 10 ± 1%, respectively – details in the SI, §12). Raman spectra obtained from a sample prepared in nearly identical conditions and transferred onto an identical TEM grid are shown in Fig. S33 of the SI. The first order spectrum contains broad and strongly overlapping D and G bands with a high D to G band intensity ratio (*I*_D_/*I*_G_) of 0.91, markedly different to that observed for pristine graphene. Following the “amorphization trajectory” described by Ferrari and Robertson,^39^ this film would be characterised as an early-to-mid “stage 2” carbon, between nanocrystalline graphite and amorphous carbon, comprising a graphitic lattice with a significant number of structural defects. Using the Ferrari and Robertson relationship (*I*_D_/*I*_G_ = *C*′(*λ*)/*L*_D_^2^), the mean inter-defect distance (*L*_D_) was estimated as 1.3 nm, which corresponds well with observations made using TEM, as shown in Fig. 5 and S28–S32 in the SI. These results confirm that this CVD method can grow films of comparable quality on Cu foils, which could potentially be transferred onto a wide array of substrates for different technological applications.

## Discussion and conclusions

We have demonstrated that topological defects in a graphene-like film can be grown with a high homogeneity of defect type by using a molecular precursor, azupyrene, that shares the topology of that defect, specifically 5-/7-membered carbon rings. Between ∼700–850 K a dendritic network is formed; between ∼850–950 K a closed film with a defect abundance of ∼20% is formed. Unlike prior work,^21,23,24^ these films are grown without potential halide contaminants and form self-limited monolayers at high coverage. The correlation of growth temperature with defect concentration permits the study of the spectroscopic details and adsorption height of the defective films as a function of the defect concentration. The NIXSW results indicate a strong link between the mean adsorption height of the film and the defect concentration, specifically the greater the defect concentration, the lower the adsorption height. Our NEXAFS measurements indicate that there may be clear spectral fingerprints related to the presence of these topological defects.

Unlike post-processing of graphene oxide^40–42^ or ideal graphene,^11–13,42–47^ which can result in significant defect inhomogeneity (*e.g.* topological, vacancy, heteroatoms and non-aromatic C defects) and Ullmann coupling methods^14–18^ that can result in significant halide contamination, the one-step CVD method with topological precursors, presented here, yields films with unprecedentedly high defect homogeneity. To the authors' knowledge, the same scale of defect homogeneity, as observed in this study, has not been reported previously.

While this work has clearly demonstrated the significant role that growth temperature plays in the retention of defects in the grown film, the role of the precursor flux and pressure has not been explored. Nucleation density, which can be controlled by varying the precursor flux,^48^ has been shown to play a significant role in the size of the resulting graphene domains.^48,49^ Obtaining control over the domain size in these topologically defective films could further minimise the inclusion of undesired defects that arise at the grain boundaries.

The maximum possible 5-/7-defect concentration in a closed two-dimensional phagraphene film^33,34^ is 40%. Note that the defect concentration of the dendritic film, shown in Fig. 2a–c, was just over 50%, which resulted in an open film. The two closed films, studied with atomic resolution in this study (one grown on Cu(111), the other on Cu foil) had a concentration of just under, and just over (respectively), 20%, roughly half of the phagraphene maximum. Thus, these films represent a comparatively high 5-/7-defect coverage in a closed two-dimensional film.

The morphology of the film (open or closed) and the defect concentration are strongly correlated and controlled by the topology of the precursor and the growth temperature. At low growth temperatures, dehydrogenative coupling dominates and dendritic films with high defect concentrations are formed by linking azupyrene molecules. At higher temperatures, Stone–Wales rearrangement anneals defects that originate from the molecules and from dehydrogenative coupling such that a closed film can form. The azupyrene precursor serves two purposes: firstly, it acts as a source of 5-/7-rings, in addition to 5-/7-rings that will be created during CVD growth. Secondly, gas-phase DFT calculations show that its non-alternant topology energetically facilitates dehydrogenative coupling when compared to the analogous alternant precursor molecule pyrene (see SI, §2) by lowering the energy of intermediate structures. Furthermore, the molecule adsorbs closer to the surface than alternant, aromatic precursors, which will increase the catalytic effect of the surface. In combination, these effects increase the rate of growth at relatively low temperatures. The fact that pristine large-domain graphene can be grown from azupyrene at 1000 K corroborates this mechanistic proposal.^27^

Sun *et al.*^50^ have previously studied the growth of graphene from a precursor with a 5-membered topology, fluorene, though *via* a solid phase deposition, rather than vapour phase as the work presented here. This work of Sun *et al.* lacks atomic resolution that would permit direct comparison to our work and, at the elevated temperatures (1025–1075 K) used in their growth, obtained largely defect free graphene (as was observed in our prior work for azupyrene on Cu(111)^27^). Fan *et al.*^34^ and Martín-Fuentes, *et al.*^51^ have used Ullman coupling of precursors with 5-membered and 7-membered topology and obtained polymer chains *via* on-surface synthesis (depositing the precursor at room temperature an then post annealing). In the work of Fan *et al.*, where a dibromo-azulene precursor was used, further successive annealing to 730 K resulted in the fusion of these polymers and the formation of 1D nanoribbons on the surface that retained the 5-membered and 7-membered topology of the initial precursor, alternated with 4-membered rings. The advantage of our CVD based process is that performing the growth at the elevated temperature induces the formation of more energetically favourable 6-membered rings which allow the film to tessellate easily in two dimensions. We suspect that other topologically interesting precursors could also be utilised in such a CVD method to grow defective graphene films that dominantly contain 5- or 7-membered rings defects. Similarly, previous work has shown that using a N-containing precursor yields N-doped graphene films,^25,50,52^ thus by utilising a N-precursor with pyrrolic or pyridinic groups, it may be possible to selectively drive the film towards pyridinic or pyrrolic defects.

Previous work has demonstrated that adsorbed molecules with a 5-membered and 7-membered topology interact more strongly with metallic substrates than their 6-membered isomers,^7,8^ which is corroborated by the NIXSW measurements presented here. Therefore, these defect sites could act as preferential anchoring sites for metallic nanoclusters for catalytic reactions. The films are thermally stable, at least at UHV conditions, and the topology also survives exposure to ambient pressure, thus films may retain the chemical and thermal stability of graphene, but with an enhanced binding to chemical species. Interacting more strongly with impinging gas molecules may provide a stronger response in the graphitic film, resulting in improved capability for sensing applications.

While the importance of defects in graphene is understood for several applications,^2,4,5^ understanding which defects play what role is an important unanswered question. In particular, understanding how the heterogeneity in the clustering of the defect sites in the grown films affect the electronic structure, both at the defect site and in the film as a whole, is an interesting question that would need to be explored. There is clear need for samples with a high defect concentration and type homogeneity that can be transferred onto technologically relevant substrates, as demonstrated here. We posit that this core methodology, of growing defective graphene films utilising a molecular precursor that shares a moiety with the defect, can be generalised to other defect types. For example: using a heteroatomic precursor could generate heteroatomic doping of the graphene film, as has been observed with nitrogen and boron containing molecules;^25,53–55^ using a precursor with a C vacancy template, could generate graphene with a high number of C vacancy sites. Potentially, designing defects into graphene layers may be as simple as synthesising molecular precursors with that signature moiety. Close-to defect-pure graphene samples can crucially support mechanistic studies in catalysis and gas sensing to optimise binding specificity and catalytic selectivity.

## Author contributions

The STM data were acquired by B. P. K, M. A. S, L. A. R. and D. A. D.; analysed and interpreted by B. P. K and D. A. D; under supervision by D. A. D. The nc-AFM data were acquired, analysed and interpreted by B. P. K., M. A. S. and J. D.; under supervision by W. A. The SXPS and NIXSW data were acquired by B. P. K., M. A. S., L. A. R., T. L., L. B. S. W. and D. A. D.; analysed and interpreted by B. P. K. and D. A. D.; under supervision by T. L. L. and D. A. D. The NEXAFS data were acquired by B. P. K., M. A. S., A. P. and D. A. D.; analysed and interpreted by B. P. K. and D. A. D.; under supervision by D. A. D.; A. G. created new software for the acquisition of the NEXAFS data. The TEM data were acquired by D. H. and C. A.; analysed by F. E. and D. A. D., with support from A. S.; and interpreted by D. A. D. The samples were grown by B. P. K., M. A. S., L. A. R., TL, L. B. S. W. and D. A. D.; under supervision by D. A. D. The azupyrene was synthesised by L. S. and S. M. W.; under supervision by G. H. The samples were transferred to TEM grids by S. S. A.; under supervision by R. G. and S. J. H. Raman spectra were measured by M. B. and G. A. R., and interpreted by G. A. R. DFT calculations were performed by B. P. K., M. A. S., D. B. M. and H. T.; interpreted by B. P. K. and R. J. M.; under supervision by R. J. M. The study was conceived and designed by B. P. K., R. J. M. and D. A. D. The paper was primarily drafted by D. A. D., with significant support from B. P. K., D. B. M., H. T., G. A. R. and R. J. M. The paper was revised by all authors.

## Conflicts of interest

The authors have no conflicts to disclose.

## Supplementary Material

SC-016-D5SC03699B-s001

## Data Availability

Data for this article, including the DFT, XPS, NIXSW, TEM, Raman and nc-AFM data are available on the Nottingham Research Data Management Repository at https://doi.org/10.17639/nott.7599. Supplementary information: additional methodological details on the sample preparation, experimental measurements and their analysis and the DFT calculations, as well as further nc-AFM, ADF-STEM and NIXSW results that are summarised in this article. See DOI: https://doi.org/10.1039/d5sc03699b.
