# Supplementary material for: One-step synthesis of graphene containing topological defects
Source: Chem Sci. 2025 Sep 9;16(41):19403–13. doi: 10.1039/d5sc03699b (PMC12459200; doi:10.1039/d5sc03699b)
Supplement: SC-016-D5SC03699B-s001 [file SC-016-D5SC03699B-s001.pdf]

Supplementary Information (SI) for Chemical Science.
This journal is © The Royal Society of Chemistry 2025

One-step synthesis of graphene containing topological defects

Benedikt P. Klein[1,2†], Matthew A. Stoodley[1,2], Joel Deyerling[3], Luke A. Rochford[1,4], Dylan B. Morgan[2], David Hopkinson[1], Sam Sullivan-Allsop[5], Henry Thake[2], Fulden Eratam[1], Lars Sattler[6], Sebastian M. Weber[6], Gerhard Hilt[6], Alexander Generalov[7], Alexei Preobrajenski[7], Thomas Liddy,[1,8] Leon B. S. Williams[1,9,10], Mhairi Buchan[1,11], Graham A. Rance[8,12] Tien-Lin Lee[1], Alex Saywell[13], Roman Gorbachev[5], Sarah J. Haigh[5], Christopher S. Allen[1,14], Willi Auwärter[3], Reinhard J. Maurer[2,15*] and David A. Duncan[1,8*]

[1]Diamond Light Source, Harwell Science and Innovation Campus, Didcot, OX11 0DE, United Kingdom.
[2]Department of Chemistry, University of Warwick, Gibbet Hill Road, Coventry, CV4 7AL, United Kingdom.
[3]Physics Department E20, TUM School of Natural Sciences, Technical University of Munich, James-Franck-Straße 1, 85748 Garching, Germany.
[4]Department of Earth Sciences, University of Cambridge, Downing Street, Cambridge, CB2 3EQ, United Kingdom.
[5]National Graphene Institute, University of Manchester, Oxford Road, Manchester, M13 9PL, United Kingdom.
[6]Institute of Chemistry, Carl von Ossietzky University Oldenburg, Carl-von-Ossietzky-Straße 9-11, 26111 Oldenburg, Germany.
[7]MAX IV Laboratory, University of Lund, Fotongatan 2, 224 84 Lund, Sweden
[8]School of Chemistry, University of Nottingham, University Park, Nottingham, NG7 2RD, United Kingdom
[9]School of Chemistry, University of Glasgow, University Avenue, Glasgow, G12 8QQ, United Kingdom.
[10]School of Physics & Astronomy, University of Glasgow, University Avenue, Glasgow, G12 8QQ, United Kingdom.
[11]School of Chemistry, University of St. Andrews, N. Haugh, St Andrews, K16 9ST, United Kingdom
[12]Nanoscale & Microscale Research Centre, University of Nottingham, University Park, Nottingham, NG7 2RD, United Kingdom
[13]School of Physics & Astronomy, University of Nottingham, University Park, Nottingham, NG7 2RD, United Kingdom
[14]Department of Materials, University of Oxford, Parks Road, Oxford, OX1 3PH, United Kingdom.
[15]Department of Physics, University of Warwick, Gibbet Hill Road, Coventry, CV4 7AL, United Kingdom.
*Corresponding authors. E-mails: r.maurer@warwick.ac.uk; david.duncan@nottingham.ac.uk;
†Current address: Research Center for Materials Analysis, Korea Basic Science Institute, 169-148 Gwahak-ro, Yuseong-gu, Daejeon 34133, Republic of Korea

Supporting Information

Table of Contents

Figure S1 3
1. Methods 3
  1.1 STM measurements 3
  1.2 SXPS measurements 3
  1.3 nc-AFM measurements 3
  1.4 NEXAFS measurements 4
  1.5 NIXSW measurements 4
  1.6 Experiments on polycrystalline Cu foils and ADF-STEM measurements 4
  1.7 SEM measurements 5
  1.8 Raman measurements 5
  1.9 Density Functional Theory Calculations 5
Figure S2 6
Figure S3 6
2. Homocoupling of azupyrene: Mechanistic considerations and Density functional theory calculations 6
Figure S4 8
Figure S5 8
Figure S6 9
Table S1 10
Figure S7 11

Figure S8-S10 ... 12
Table S2 ... 14
Figure S11 ... 14
3. Sources of asymmetrical loss features in X-ray photoelectron spectroscopy ... 14
4. XPS measurements excluding contaminants and indicating self-limiting growth ... 14
Figures S12-S14 ... 15
Figure S15 ... 17
5. Further discussion on the experimental NEXAFS data ... 17
6. Details on the computed XPS and NEXAFS data: Model structures ... 17
7. Details on the computed XPS and NEXAFS data: XPS calculations ... 18
8. Details on the computed XPS and NEXAFS data: NEXAFS calculations ... 18
Figure S16 ... 18
9. Shifting of the binding energy and photon energy scales in the calculated spectra ... 19
Figure S17 ... 19
10. Normal incidence X-ray standing waves ... 19
11. Calibration of sample growth temperature ... 20
Figure S18 ... 21
Figures S19-S25 ... 22
12. Further details on the ADF-STEM measurements ... 28
Figure S26 ... 28
Figure S27 ... 28
Figures S28-32 ... 30
Figure S33 ... 34

Figure S1

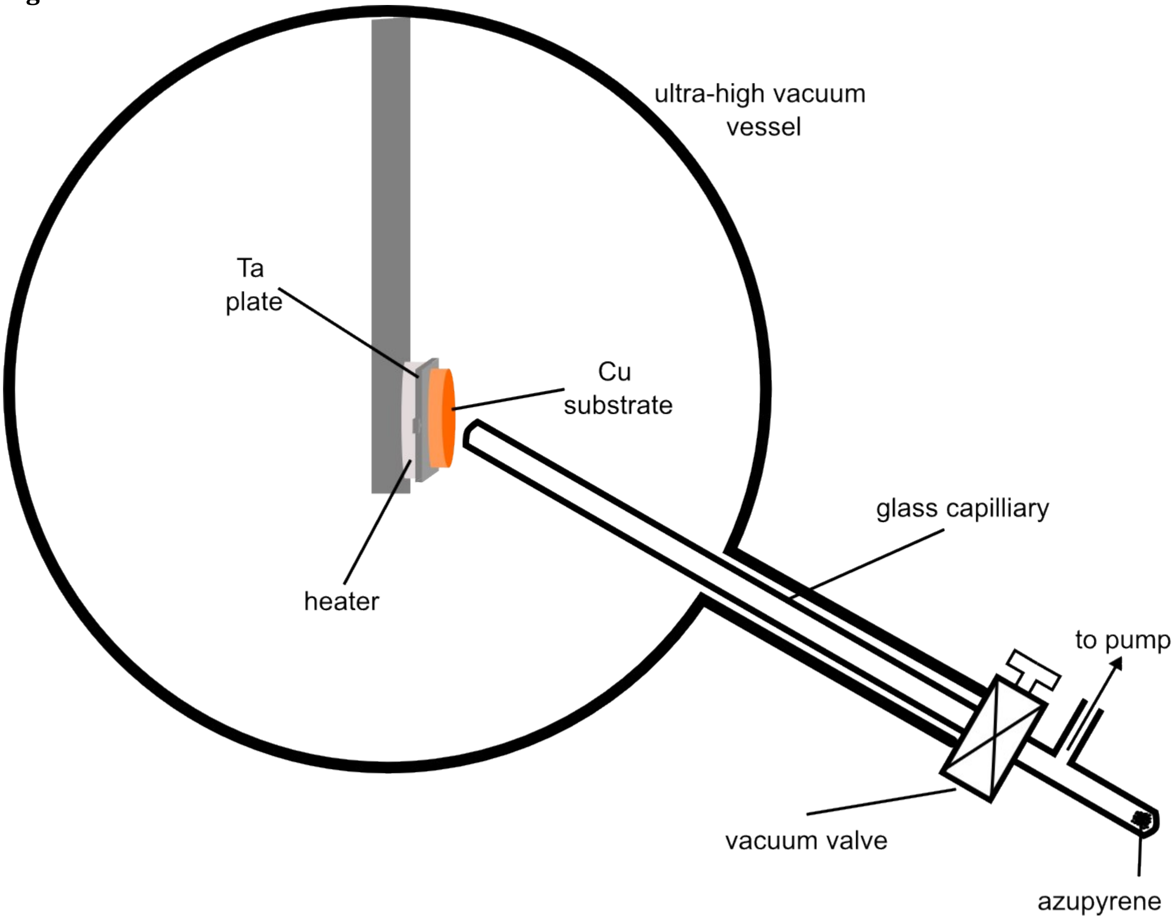

Figure S1: Generalised schematic of the growth geometry used in this study. A Cu sample, either a single crystal with a (111) termination, or a Cu foil was loaded into a receptor in the ultra-high vacuum (UHV) growth vessel. The receptor would have the facility for annealing samples to temperatures exceeding 1000 K, and the vessel would have the facility for sputtering the samples for cleaning. Our home-built evaporator is discussed in greater detail in the supporting information of Ref. [1]. This evaporator, when the vacuum valve was open, would provide direct line of sight between the azupyrene evaporant and the sample surface via a glass capillary. The test-tube containing azupyrene was differentially pumped down to a pressure of $\sim 10^{-6}$ mbar, assuring the purity of the azupyrene.

1. Methods

1.1 STM measurements

STM measurements were performed at the Surface Interface Laboratory (SIL) at the Diamond Light Source (DLS) using an Omicron VT STM with an etched tungsten tip at room temperature. The base pressure in the chamber was 5×10^{-10} mbar and samples were prepared in situ in the same vacuum system. STM measurements were performed using an Omicron VT STM

1.2 SXPS measurements

SXPS measurements were performed at the I09 beamline at the synchrotron Diamond Light Source.[2] Samples were either prepared in situ in the same vacuum system or transferred from the SIL preparation chamber via vacuum transfer. The SXPS data was recorded using the synchrotron light utilising a Scienta EW4000 HAXPES hemispherical electron energy analyser mounted perpendicular to the incident direction of the X-ray radiation in the same plane as the photon polarisation (linear horizontal). A photon energy of 430 eV was used for the C 1s spectra, the binding energy was corrected by performing a Fermi energy (FE) measurement at the same photon energy and pass energy, and setting the FE to be the origin of the binding energy scale.

1.3 nc-AFM measurements

nc-AFM measurements were performed at the Physics Department of the Technical University of Munich (TUM) using a commercial 4K LT-STM / nc-AFM Createc microscope, operated at approximately 6 K during

measurements. This microscope contains a qPlus sensor[3] (frequency-modulation mode at an oscillation amplitude of 60 pm) for performing the nc-AFM measurements, which were performed in constant high mode (open feedback loop) at sample bias of approximately 0 V. Samples were prepared in situ in the same vacuum system. For reliable / easier tip functionalisation, NaCl islands were grown (sample temperature -10 to 10 °C) after the initial sample preparation with azupyrene. The NaCl grew predominately on graphene-free Cu(111) areas. CO was dosed onto the cold sample (< 10 K) and the tip was functionalised by picking up CO from the NaCl islands. Note that the films measured in this study were highly corrugated, nc-AFM measurements only work over a relatively narrow band of adsorption heights for a given tip height above the surface. In general this is a particular struggle when measuring the adsorption structure of non-planar molecules on surfaces, e.g. Ref. [4]. The nc-AFM measurements of the dendritic and defective film were each performed on a single sample over multiple spots on those two samples. The abundance of 5-, 6- and 7- membered rings was determined by Voronoi tessellation. The centre of the ring, used as the Voronoi seed, was identified manually. Using the centre of the ring as a Voronoi seed, rather than directly attempting to draw the bonds on the measured microscopic data yields a significant advantage in that identifying the centre of a ring is far less ambiguous than locating the vertices (and thus the atoms) in the images. Areas of the images that were deemed too challenging to identify these positions were masked (e.g. the region circled in red in Figure S6 in the SI) and the resulting Voronoi diagram was composed of the unmasked regions. The MATLAB built in voronoi command, inputted with the identified position of the ring centres in the image, was used to create the Voronoi diagram and provided the vertices of the rings and thus its associated topology. The counting of the data was assumed to be Poisson distributed and thus the uncertainty in the enumerated number of rings of each topology was assumed to be the square root of the number of counted rings.

1.4 NEXAFS measurements

NEXAFS measurements were recorded in partial electron yield (PEY) mode at the FlexPES beamline at the synchrotron MAX IV.[5] The PEY detector was a multi-channel plate (MCP) mounted below the sample. A retardation grid was mounted on the MCP to act as a high-pass filter, rejecting low energy electrons, resulting in improved surface sensitivity.[6] For the measurements presented here a retardation bias of 150 V was used. Each spectrum was measured between 5-10 times across different spots on the sample. These data were integrated together and then normalised by the drain current from a clean gold monitor, mounted between the photon source and the sample. A comparable spectrum was measured from the clean sample and this "crystal spectrum" was then normalised to have the same intensity in the pre-edge region 275.0-282.5 eV as the experimental spectrum. The crystal spectrum was then subtracted from the experimental spectrum and the resulting spectrum was normalised to the average intensity over the photon energy range of 320-325 eV.

1.5 NIXSW measurements

NIXSW measurements were performed at the I09 beamline at the synchrotron Diamond Light Source. Samples were either prepared *in situ* in the same vacuum system or transferred from the SIL preparation chamber via vacuum transfer. The measurement setup for the photoelectron yield was the same as for the regular XPS data, due to the requirements of NIXSW, the hard X-ray branch of I09 was used. The reflectivity was acquired simultaneously to the photoelectron yield using a fluorescent plate that is mounted inside the flange through which the incident photon pass. The measured reflectivity curve was used to define the position of the Bragg energy, as well as the broadening present in the system due to imperfections in the monochromator or the Cu(111) single crystal. The non-dipolar effects in the NIXSW measurement[7] were addressed using a so-called "backwards-forwards Q-parameter",[8] which was calculated theoretically[9] using the angle between photon polarisation and the median photoelectron intensity emission angle (θ = 18°). The individual energy distribution curves of the NIXSW measurements were fitted with a convolution of a Gaussian and a Doniach-Sunjic line shape[10]; and a Gaussian error function, that shared the same Gaussian width as the peak, was used to model the step in the photoelectron intensity before and after the peak. A straight line was used to model the background variation. The area of the fitted peak was then used as the photoelectron yield and plotted relative to their off-Bragg photoelectron yield.

1.6 Experiments on polycrystalline Cu foils and ADF-STEM measurements

The defective graphene sample on polycrystalline Cu foil (Advent Research Materials, 99.996+% purity, 0.025 mm thick) was grown in-situ in the sample preparation chamber of the I09 beamline with pressure in the low 10^{-10} mbar range. The Cu foil was prepared by repeated sputter (V = 1 keV, p = 2×10^{-5} mbar Ar) and annealing (T = 1000 K) cycles. The cleanliness of the foil was assessed by XPS. After growth (~700 K) and XPS measurements, the foil was removed from vacuum and transported in an inert atmosphere to a clean room at the National Graphene Institute in Manchester. The defective graphene film was transferred

to the TEM grid using the below procedure. The copper foils were spin-coated (3000 rpm, 1 min) with PMMA/Anisole solution (8%) sample side up, and subsequently set on a hot plate (130 °C, 5 min). Two pieces of tape, with holes in the centre, were attached to the PMMA coated foil. The foils were submerged in the APS solution for 21 hours to allow complete etching of the copper. Each sample was then transferred to a silicon nitride TEM grid via a transfer rig. Once attached, the TEM grid was then heated to 75 °C to adhere the sample to the grid and the tape removed. The TEM grid was then heated to 130 °C on a hot plate for 5 mins. Finally, the PMMA was removed by being stirred in acetone (50 °C, 5 min) before subsequently being dipped in acetone (RT), warm IPA (50 °C), allowed to dry and then dipped in toluene (RT). The optical microscopy measurements were performed using a Nikon camera, the scale bar is referenced with respect to the grid in the centre, which is 150 μm wide.

ADF-STEM measurements were performed using a JEOL ARM300CF microscope operating at 80 keV with convergence semi-angle of 25 mrad, ADF collection angular range of 47 to 170 mrad and a beam current of approximately 30 pA. The images were filtered by 2D-Gaussian filtering (MATLAB 2023a, imgausfilt) whose two dimensional smoothing kernel had a standard deviation of 3.5 pixels. The abundance of 5-, 6- and 7- membered rings was determined by Voronoi tessellation using the same procedure as detailed in the nc-AFM section.

1.7 SEM measurements

SEM images were acquired using a FEI (Thermo Fisher) Quanta 650F field emission gun scanning electron microscope (FEG-SEM). Samples were sufficiently conductive for imaging without further coating. Typical accelerating voltages were between 20 and 30 kV.

1.8 Raman measurements

Micro Raman spectroscopy was performed using a HORIBA LabRAM HR Evo Raman spectrometer. Spectra were collected using a 532 nm laser (at ~0.3 mW power), a 100x objective and a 100 µm confocal pinhole. To simultaneously scan a range of Raman shifts, a 600 lines mm^{-1} rotatable diffraction grating along a path length of 800 mm was employed. Spectra were acquired using a Synapse EMCCD detector (1024 pixels) thermoelectrically cooled to −60 °C. Before spectra collection, the instrument was calibrated using the zero-order line and a standard Si(100) reference band at 520.7 cm^{-1}. The spectral resolution in this configuration is better than 1.1 cm^{-1}. The samples measured by Raman spectroscopy were prepared following the same recipe as that described in §1.6.

1.9 Density Functional Theory Calculations

Density Functional Theory (DFT) calculations have been performed with the all-electron atomic-orbital code FHI-aims (v210716)[11] and the pseudopotential plane-wave code CASTEP[12] (version 21.1) using the PBE exchange-correlation functional[13] with the vdW^{surf} dispersion correction[14] for the NEXAFS simulations in Figure 3, and the PBE+vdWsurf and M06-2X[15] functionals for the study of the dehydrogenative coupling mechanism reported in §2 of the ESI. FHI-aims and the PBE+vdW^{surf} functional were used for all geometry optimizations. Optimized structural models of freestanding ideal graphene, Cu(111)-adsorbed ideal graphene, freestanding Stone-Wales-defective graphene and Cu(111)-adsorbed Stone-Wales defective graphene are based on previous work[16] and built from a Moiré superstructure of a $3\sqrt{13} \times 3\sqrt{13}$ Cu(111) surface slab that accommodates a $2\sqrt{31} \times 2\sqrt{31}$ graphene layer with an angle of rotation of 5°. CASTEP with standard on-the-fly generated ultrasoft pseudopotentials was used to perform XPS and NEXAFS simulations for the optimized structures. XPS simulations were performed with the Delta-Self-Consistent-Field (Δ-SCF) method and NEXAFS simulations were performed with the ionisation-potential-corrected transition-potential method (ΔIP-TP).[17-19] Spectra were broadened with a pseudo-Voigt function to capture instrumental and lifetime broadening effects.[20] For the SXPS modelling, a Gaussian full width half maximum (FWHM) of 0.40 eV and a Lorentzian FWHM of 0.46 eV were used. The NEXAFS spectra were broadened with a pseudo-Voigt function with an energy-dependent broadening model, starting with a 0.75 eV broadening at an 80%/20% Gaussian-Lorentzian ratio at the leading edge. Further details on DFT calculations for the dehydrogenative coupling mechanism and core-level spectra simulations are given in §2 and §5-7, respectively.

Figure S2

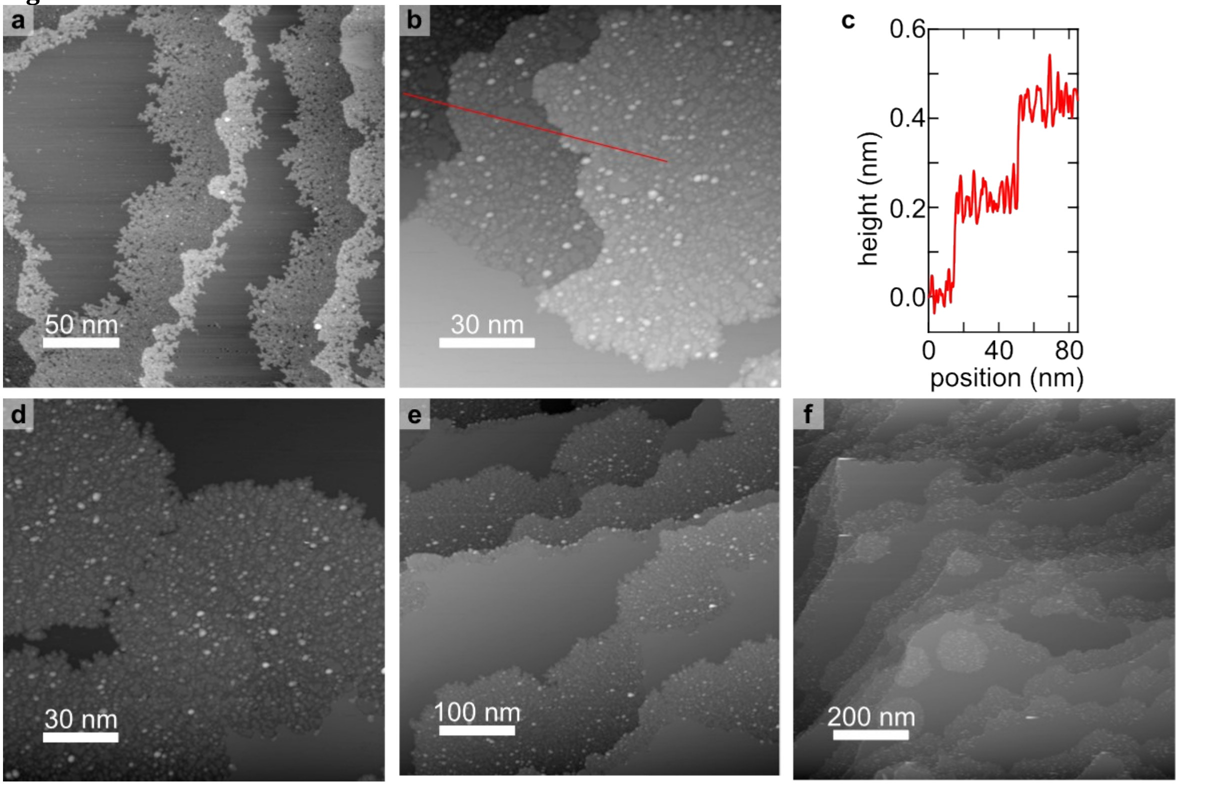

Figure S2. STM measurements of the (**a**) dendritic and (**b**,**d-f**) defective films shown in Figure 1 of the main manuscript in different areas over longer length scales. [STM parameters: (**a**) U_{tip} = +1.5 V, I_{tunnel} = 1.25 nA; (**b**, **d-f**) U_{tip} = -1.9 V, I_{tunnel} = 0.5 nA]. Also shown (**c**) is a line-profile taken across two steps in the island shown in panel (**b**), indicated by a red line in panel (**b**). The steps, along the profile, have an approximate height of 0.2 nm, corresponding well to the step height of the Cu(111) surface, 0.2087 nm. These STM data demonstrate a sub-monolayer growth of the dendritic and defective films and indicate no evidence of the growth of second or higher layer growth on the surface.

Figure S3

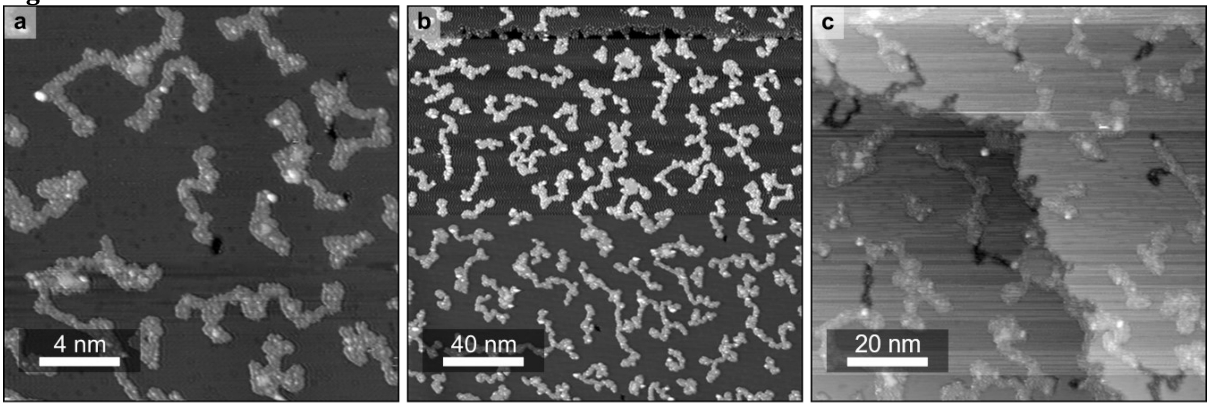

Figure S3. STM measurements of a saturated monolayer of azupyrene deposited onto Cu(111) and then post annealed to (**a,b**) ~725 K and (**c**) ~820 K. [STM parameters: (**a,b**) U_{tip} = -2.0 V, I_{tunnel} = 2.5 nA; (**c**) U_{tip} = -3.5 V, I_{tunnel} = 1.0 nA].

2. Homocoupling of azupyrene: Mechanistic considerations and Density functional theory calculations

In the case of a simple homocoupling of two azupyrene molecules there are three symmetrically unique bonding configurations, as shown in Figure S4. Two configurations result in a 5-membered ring being formed, one results in a 6-membered ring. There are 16 possible combinations of the configurations in Figure S4a that yield a 5-membered ring and the configuration in Figure S4c that yields a 6-membered ring, but only 8 combinations that will yield the configuration in Figure S4b. Thus, by considering this first linking stage, one would naively expect homocoupling to result in 50% more 5-membered rings, than 6-membered, rings. Regardless of the actual ratio of 5- to 6-membered rings found to link these molecules,

that 7-membered rings cannot form in this way suggests why in both dendritic and defective films 5-membered rings are found to be more numerous.

Exploring the mechanism of this simplistic dehydrogenative coupling further, we performed gas-phase DFT calculations into the energetics of dimerising azupyrene molecules and pyrene molecules. While these calculations neglect the role of the hot surface in catalysing the dehydrogenation and the homocoupling of molecules, they provide some indication over which of the possible bonding configurations shown in Figure S4 are thermodynamically favourable and whether or not a difference exists between pyrene and azupyrene in terms of the growth dynamics.

All calculations were performed with FHI-aims employing the 'tight-tier2' basis set with the internal default tight settings for the integration grid and basis functions. Convergence of geometry optimisations was performed using the Broyden-Fletcher-Goldfarb-Shanno optimisation algorithm until a maximum residual force component per atom of 5×10^{-3} eV Å^{-1} was obtained while the electronic convergence criterion was set to 1×10^{-5} e Å^{-3} for electron density. Relativistic effects were accounted for using the atomic ZORA functionality, with higher-order effects neglected for the studied atomic environments. Multiple exchange correlation functionals were used, namely the PBE+vdW^{surf} and the M06-2X functionals.

We have determined the energy barrier for Stone-Wales transformation (SWT) between pyrene and azupyrene using the climbing image nudged elastic band (NEB) method implemented the ASE code[21] to a global force tolerance of 0.05 eV Å^{-1}. To provide transferability and comparison to on-surface simulations, the PBE+vdW^{surf} functional was initially employed, from which the C' dimer (see Figure S4) was re-evaluated using the more accurate hybrid functional, M06-2X, for the description of bond breaking events[22]. The corresponding barriers are 6.04 (6.72) and 7.91 (9.09) eV for conversion from azupyrene to pyrene or pyrene to azupyrene, respectively. Numbers are given for PBE+vdW^{surf} and M06-2X (the latter in parentheses).

For the dehydrogenative coupling, the energies of intermediate states upon dehydrogenation and C-C homocoupling were calculated by structural relaxation after removal of a hydrogen atom. Where the number of hydrogen atoms changes in the molecule, reactions are balanced by accounting for the energy of a hydrogen molecule or ½ of hydrogen molecule. The entire reaction scheme is shown in Figure S5 and the individual reaction equations of the corresponding chemical transformations that are considered are listed below with the different species indicated with roman numerals:

$$C_{16}H_{10}(pyrene, I) \rightarrow C_{16}H_{10}\ (azupyrene, II), \quad \textbf{(1)}$$

$$C_{16}H_{10}(I\ or\ II) \rightarrow {}^{\bullet}C_{16}H_9 + \frac{1}{2}H_2, \quad \textbf{(2)}$$

$$C_{16}H_{10} + {}^{\bullet}C_{16}H_9 \rightarrow C_{32}H_{18}\ (III\ or\ IV) + \frac{1}{2}H_2, \quad \textbf{(3)}$$

$$C_{32}H_{18}\ (III\ or\ IV) \rightarrow {}^{\bullet}C_{32}H_{17}\ (V\ or\ VI) + \frac{1}{2}H_2 \quad \textbf{(4)}$$

$${}^{\bullet}C_{32}H_{17}\ (V\ or\ VI) \rightarrow C_{32}H_{16}\ (VII\ or\ VIII) + \frac{1}{2}H_2, \quad \textbf{(5)}$$

$$C_{32}H_{16}\ (VII\ or\ VIII) \rightarrow C_{32}H_{16}\ (IX), \quad \textbf{(6)}$$

where reaction **(1)** is the SWT, reaction **(2)** is the removal of a single hydrogen from one of the sites of pyrene (I) or azupyrene (II) described in Figure S4, reaction **(3)** is the carbon-carbon bond formation and simultaneous removal of a hydrogen atom to form a single-bonded pyrene dimer (III) or azupyrene dimer (IV), reaction **(4)** is the removal of a single hydrogen atom from the carbon adjacent to the newly formed dimer bond, forming pyrene (V) and azupyrene (VI) dimer radicals, reaction **(5)** is **(5a)** the formation of the 2nd C-C bond and **(5b)** removal of another hydrogen to form a pyrene (VII) or azupyrene dimer (VIII). Finally, reaction **(6)** is the conversion of a pyrene dimer or azupyrene dimer, via a single SWT, to a pyrene-azupyrene dimer (IX). All reactions and structures are depicted in the reaction diagrams in Figures S5 and S6 with reaction energies given by PBE+vdW^{surf} and M06-2X, respectively.

The kinetic and thermodynamic processes in Figure S5 are depicted graphically in Figure S6 and summarized in Table S1. Figure S6 shows that the C/C' dimerization geometry is the most energetically

favourable coupling in terms of the reaction cascade shown in Figure S5, although energy differences are rather small. We have therefore repeated the calculations for C/C' dimerization with the M06-2X functional. From the calculated energetics and barriers, we can conclude that barriers for Stone-Wales transformation are significantly larger than the highest barriers for dehydrogenative coupling of two molecules. As a result, at elevated surface temperatures, dehydrogenative coupling will dominate SWT. This is true for pyrene and azupyrene molecules. While the initial dehydrogenation is energetically similar for pyrene and azupyrene, the second dehydrogenation that forms the final 6-ring that connects the dimer is energetically far more favourable for azupyrene dimerization than for pyrene dimerization. This arises from the instantaneous formation of a single bond in structure VI, which is shown as the additional reaction **(5a)** step in the azupyrene dimerization, in Figure S5, preceding the reaction **(5b)** step. This additional bond formation leads to re-hybridisation of the newly-bonded-to carbon and delocalisation of the radical into the altered π-system. Therefore, in addition to evidence for azupyrene motifs being retained during dimerization, we find that the dimerization of non-alternant azupyrene molecules provides an energetic advantage over the dimerization of alternant pyrene molecules.

Figure S4

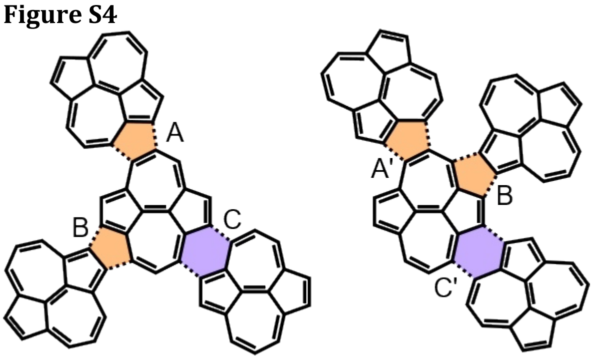

Figure S4: Possible bonding configurations of two azupyrene molecules. Solid lines indicate bonds within the azupyrene molecules, hashed lines indicate possible bonds formed by dehydrogenative homocoupling of the molecules. As shown there are two possible configurations that result in a 5-membered ring (A / A' and B) and one configuration that results in a 6-membered ring (C / C'). In this simplified two molecule case, there is no possible configuration that yields a 7-membered ring.

Figure S5

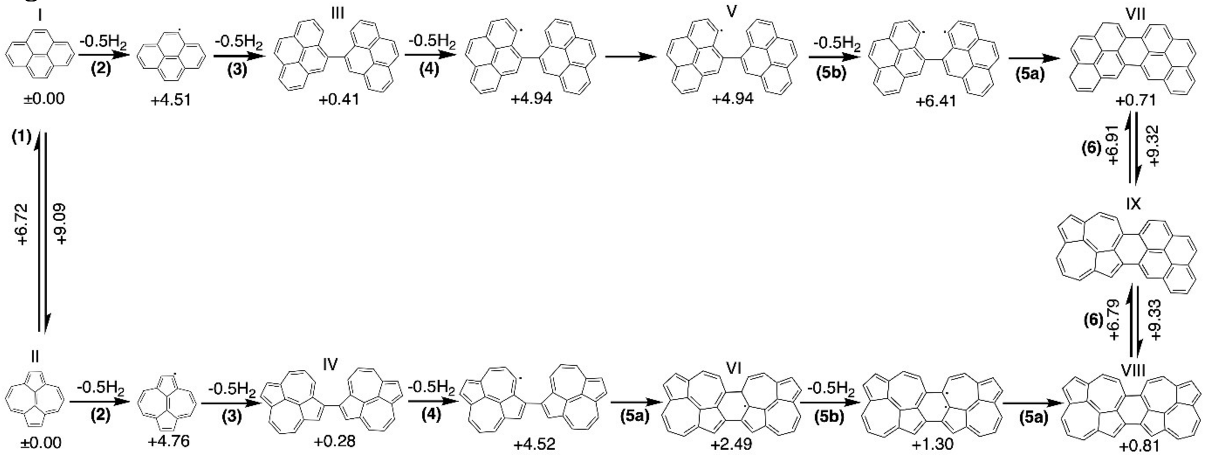

Figure S5: Reaction scheme for the C/C' dehydrogenative dimerization of pyrene and azupyrene and the Stone Wales transition of pyrene (I) and azupyrene (II) as well as their dimers. Roman numerals indicate structures, and the individual reactions are labelled with their associated reaction equation number detailed in the text of §2 of the SI. The relaxed M06-2X gas-phase energies for each species are given in eV. The values above arrows on reactions **(1)** and **(6)** correspond to gas-phase reaction barriers in eV.

Figure S6

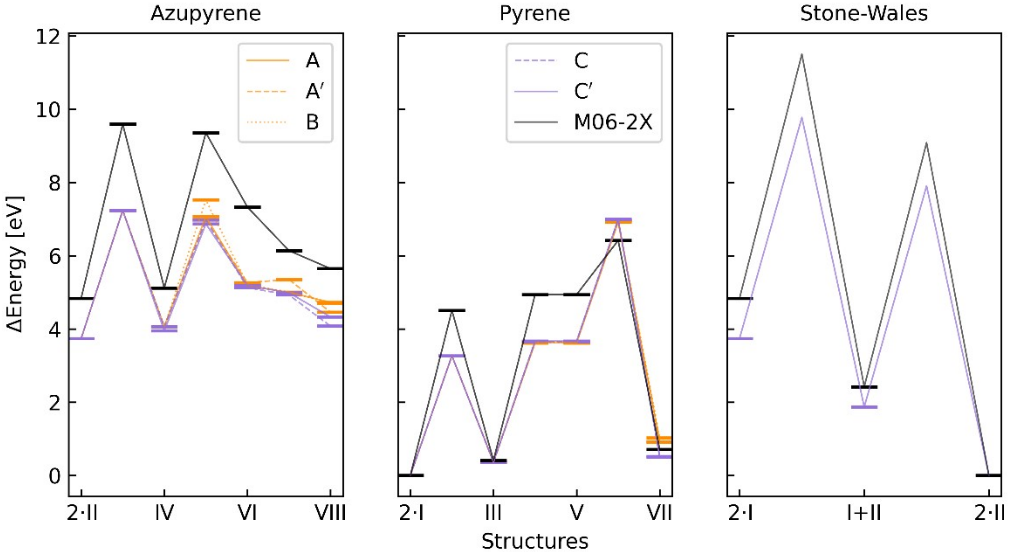

Figure S6: Energy level diagram for reaction steps of gas-phase dehydrogenative coupling of azupyrene and pyrene. Gas-phase energies predicted with PBE+vdW^{surf}, unless otherwise indicated. The tabulated values can be found in Table S1. All shown values are relaxed intermediate values, except for barriers shown in the right-hand panel, which correspond to the first order transition states. The indicated A, A', B, C and C' diagrams relate to the final products of the reaction, shown in Figure S4.

Table S1

Table S1: Gas-phase kinetic barriers ($\Delta E^{\ddagger}$) and reaction energies (ΔE) for the stated processes shown in Figure S5. The energies here are represented graphically in Figure S6. The reaction energies are detailed for each of the possible product structures shown in Figure S4. The kinetic barriers correspond to the lowest overall barrier process for each structure calculated using the PBE+vdW^{surf} (M06-2X) functional. Only processes for C' were calculated with M06-2X. For reaction **(5a)** (azupyrene) the reaction energies of the single and double bond components are summed together for direct comparison to reaction **(5a)** (pyrene), where these values cannot be separated for the single-step process. For reaction **(6)** the first energy corresponds to the VII-IX barrier, the second to the VIII-IX barrier.

Reaction	Product	Kinetic Barrier ($\Delta E^{\ddagger}$ - eV)		Reaction Energies (ΔE - eV)	
		Azupyrene	Pyrene	Azupyrene	Pyrene
**(1)**	--	+7.91 (+9.09)	+6.04 (+6.72)	-1.87 (-2.42)	1.87 (2.42)
**(2)**	A	--	--	+3.50	+3.27
	A'			+3.50	+3.27
	B			+3.50	+3.27
	C			+3.50	+3.27
	C'			+3.50 (+4.76)	+3.27 (+4.51)
**(3)**	A	--	--	-3.17	-2.90
	A'			-3.18	-2.91
	B			-3.18	-2.91
	C			-3.18	-2.91
	C'			-3.29 (-4.48)	-2.90 (-4.10)
**(4)**	A	--	--	+3.00	+3.25
	A'			+2.92	+3.30
	B			+3.47	+3.27
	C			+2.92	+3.30
	C'			+2.92 (+4.24)	+3.30 (+4.52)
**(5a)**	A	--	--	-2.16	-6.00
	A'			-2.60	-6.03
	B			-2.62	-5.93
	C			-2.70	-6.49
	C'			-2.34 (-2.52)	-6.48 (-5.72)
**(5b)**	A	--	--	-0.18	+3.30
	A'			+0.08	+3.29
	B			-0.20	+3.33
	C			-0.20	+3.33
	C'			-0.20 (-1.19)	+3.33 (+1.48)
**(6)**	A	+6.09, +6.18	+8.02, +8.25	-1.93, -2.07	+1.93, +2.07
	A'	+6.06, +6.83	+8.02, +8.58	-1.96, -1.75	+1.96, +1.75
	B	+6.26, +6.08	+8.18, +8.02	-1.82, -1.94	+1.82, +1.94
	C	+6.26, +6.40	+8.22, +8.21	-1.96, -1.81	+1.96, +1.81
	C'	+6.30, +6.10 (+6.91, +6.79)	+8.19, +8.13 (+9.32, +9.33)	-1.89, -2.03 (-2.41, -2.54)	+1.89, +2.03 (+2.41, +2.54)

Figure S7

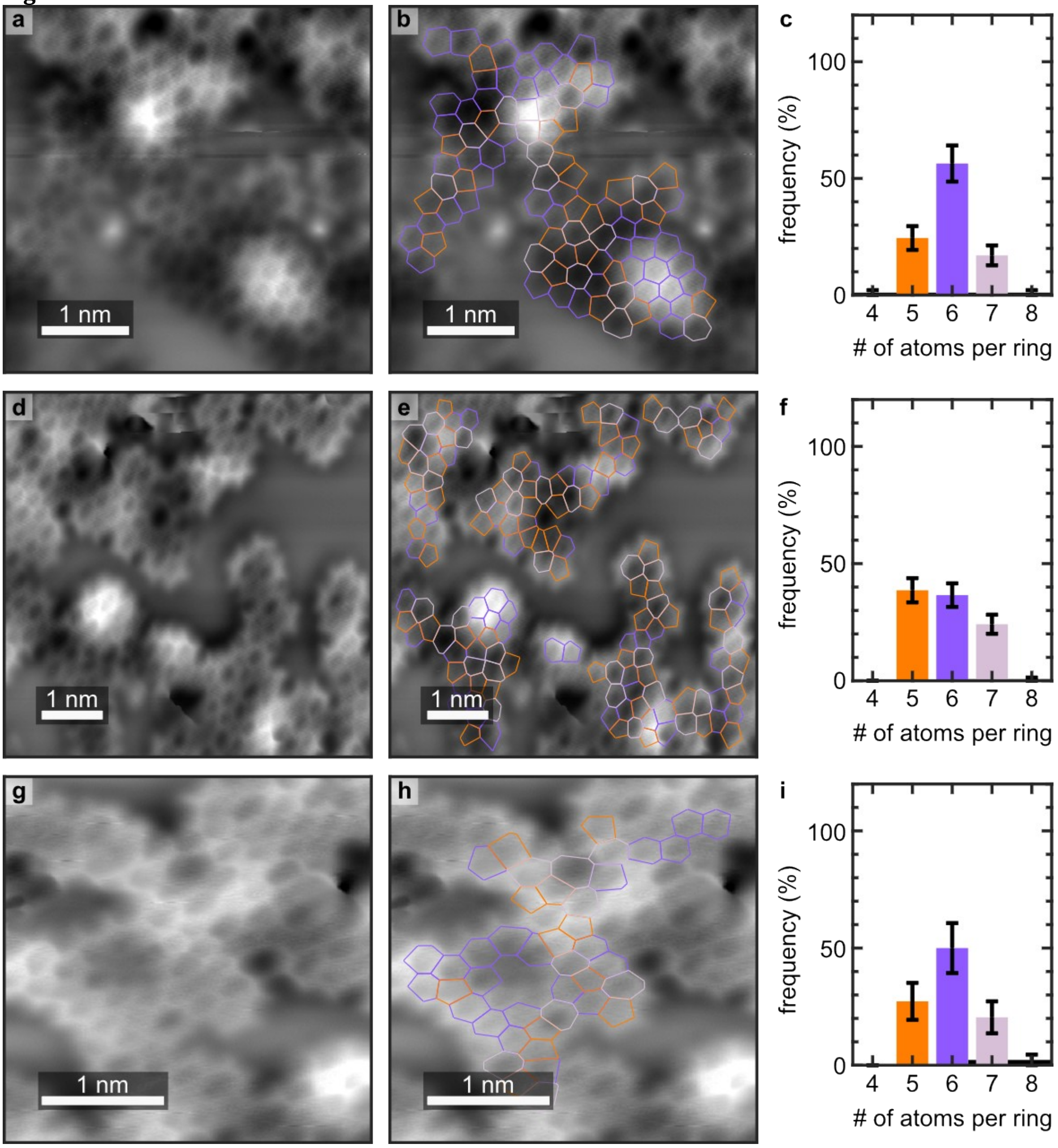

Figure S7: (**a**-**b**,**d**-**e**,**g**-**h**) Atomically resolved nc-AFM measurements of a dendritic film. Overlayed in panels (**b**,**e**,**h**) are the C-C bonds, 5-membered rings are dark orange, 7-membered are light purple and 6-membered are dark purple. Also shown in panels (**c**,**f**,**i**) are the histograms of the relative frequency of 4- to 8-membered rings. Imaging conditions are stated in the methods section of the main article.

Figure S8-S10

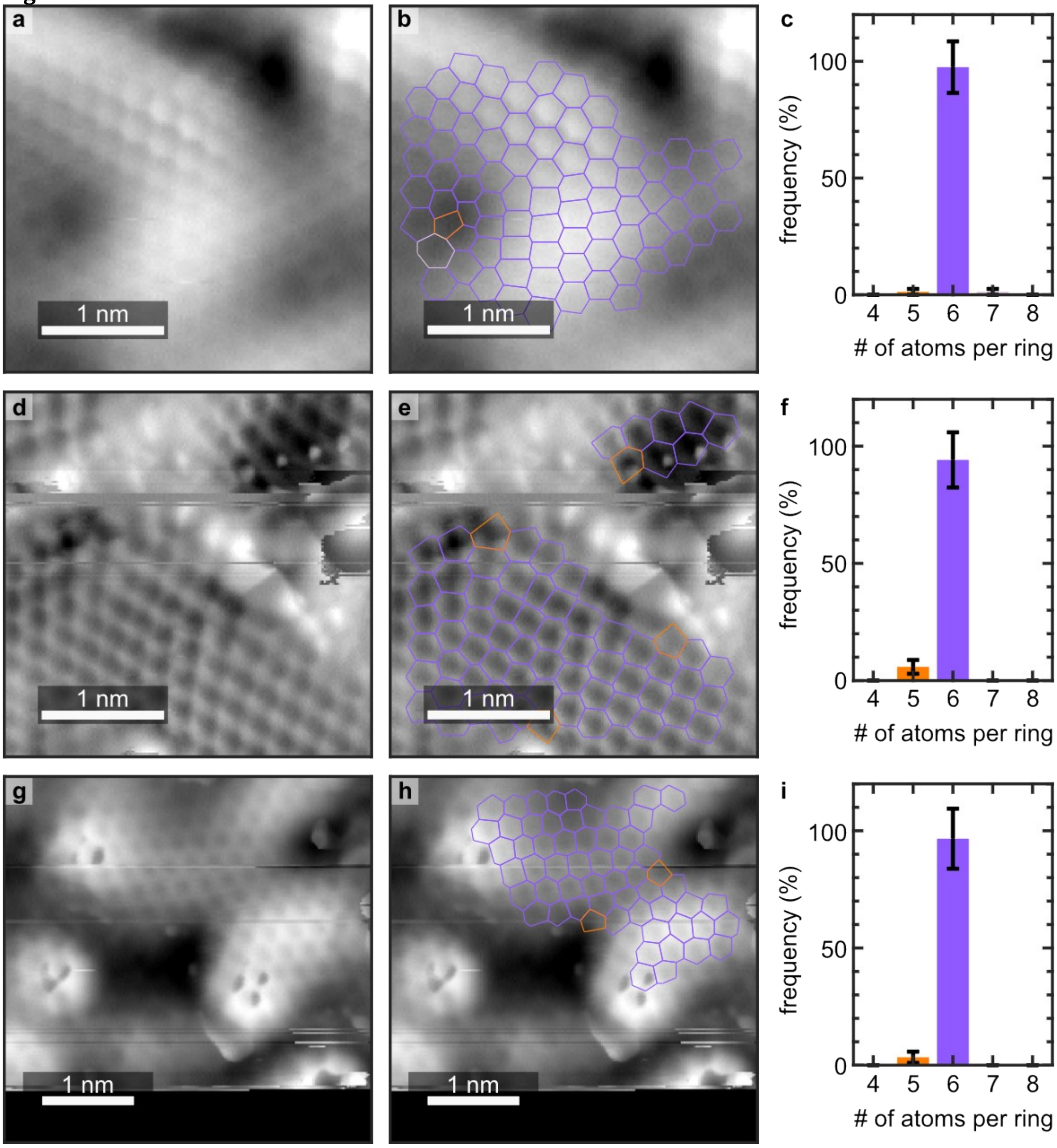

Figure S8: (**a**-**b**,**d**-**e**,**g**-**h**) Atomically resolved nc-AFM measurements of a defective film. Overlayed in panels (**b**,**e**,**h**) are the C-C bonds, 5-membered rings are dark orange, 7-membered are light purple and 6-membered are dark purple. Also shown in panels (**c**,**f**,**i**) are the histograms of the relative frequency of 4- to 8-membered rings. Imaging conditions are stated in the methods section of the main article.

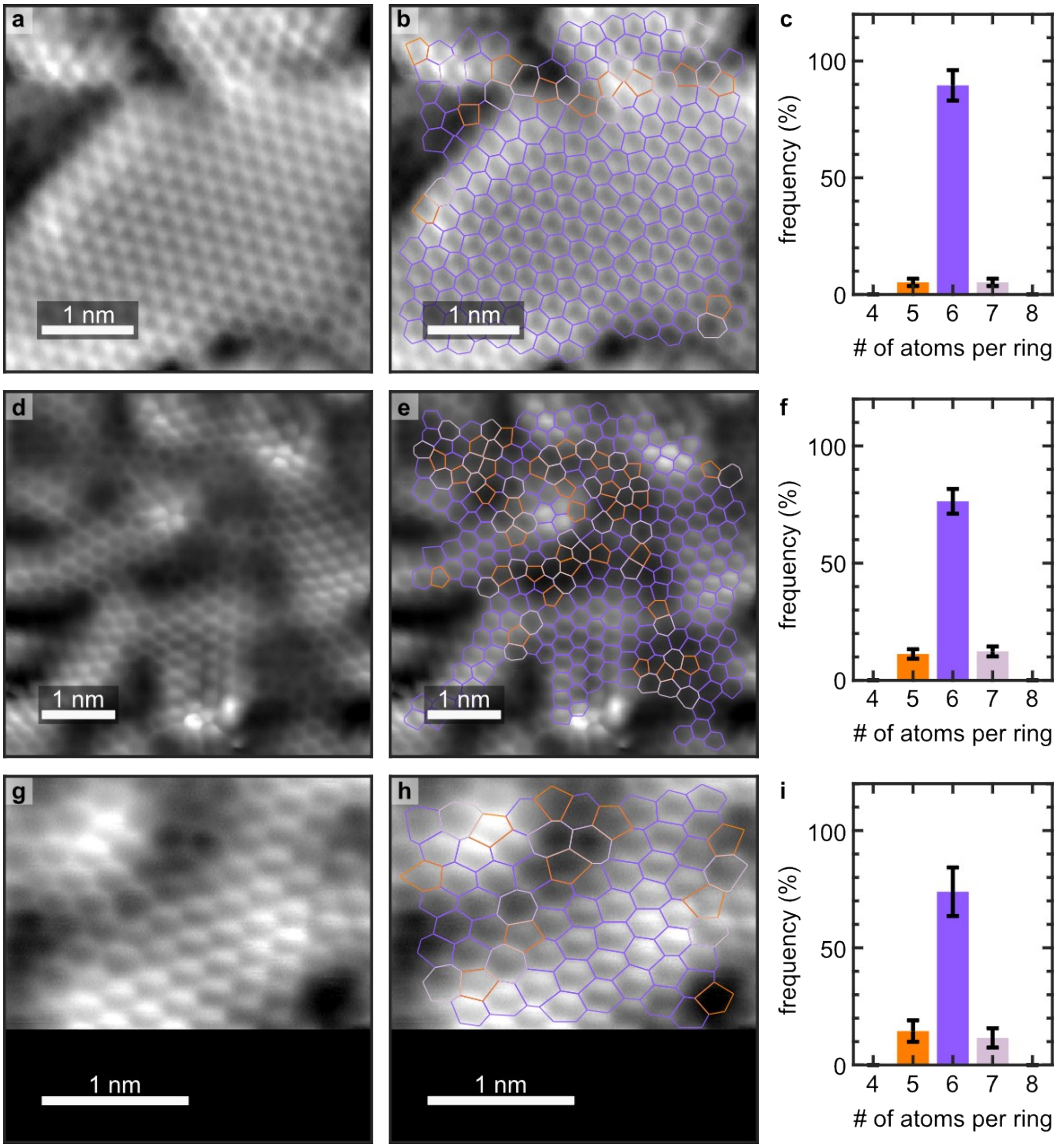

Figure S9: (**a**-**b**,**d**-**e**,**g**-**h**) Atomically resolved nc-AFM measurements of a defective film. Overlayed in panels (**b**,**e**,**h**) are the C-C bonds, 5-membered rings are dark orange, 7-membered are light purple and 6-membered are dark purple. Also shown in panels (**c**,**f**,**i**) are the histograms of the relative frequency of 4- to 8-membered rings. Imaging conditions are stated in the methods section of the main article.

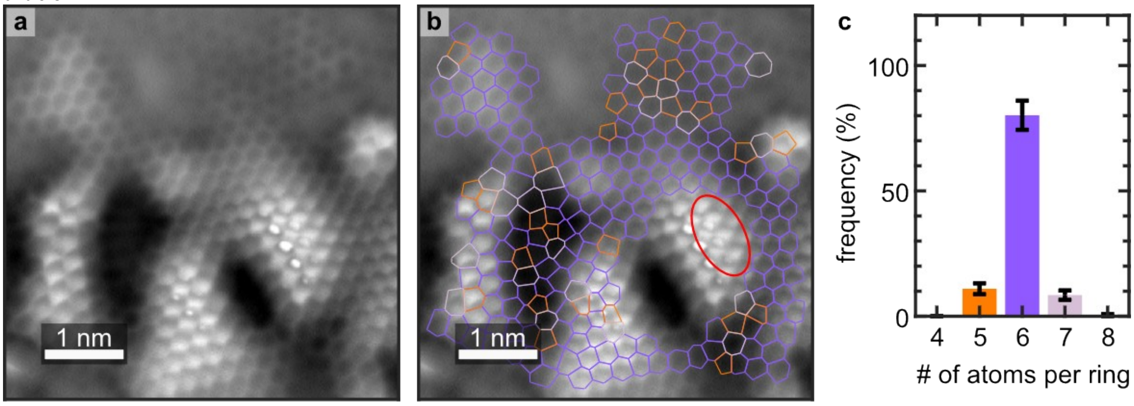

Figure S10: (**a**,**b**) Atomically resolved nc-AFM measurements of a defective film. Overlayed in panel (**b**) are the C-C bonds, 5-membered rings are dark orange, 7-membered are light purple and 6-membered are dark purple. Also shown in panel (**c**) is the histograms of the relative frequency of 4- to 8-membered rings. Imaging conditions are stated in the methods section of the main article. Circled in red are a row of six membered rings that transition from the centre of the ring contrasting darkly, to the centre of the ring contrasting brightly, hindering local assignment of the topology.

Table S2

Table S2: The enumeration, both in raw number and percentage, of the number of rings observed that are 4- to 8-membered for the defective and dendritic films measured by nc-AFM, as well as the defective film measured by ADF-STEM. The value in brackets is the number of rings identified with that number of atoms / bonds. The error, in percentage, for the nc-AFM: dendritic sample is 6%, nc-AFM : defective is 3% and ADF-STEM : defective is 1%.

technique	film	4-	5-	6-	7-	8-
nc-AFM	dendritic	0% (1)	32% (91)	45% (128)	21% (60)	1% (3)
	defective	0% (0)	9% (85)	84% (839)	7% (74)	0% (1)
ADF-STEM	defective	0% (11)	11% (726)	78% (5101)	10% (639)	0% (28)

Figure S11

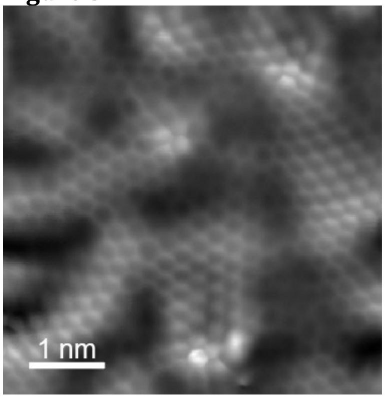

Figure S11: Reproduction of the nc-AFM measurement shown in Figure 2d of the main manuscript with a color scale that covers the whole range of measured heights. Imaging conditions for the nc-AFM measurements are in the Methods.

3. Sources of asymmetrical loss features in X-ray photoelectron spectroscopy

The soft X-ray photoelectron spectroscopy (SXPS) obtained from molecular azupyrene and graphene grown on Cu(111) are shown in Figure 3a of the main manuscript. The graphene C 1s SXPS is found at a higher BE than the molecular azupyrene and consists of a single, narrow peak, indicating closely similar chemical environments for each carbon atom. The C 1s SPXS of molecular azupyrene exhibits a shoulder at lower BE, that has previously been discussed in detail.[1] The C 1s SXPS spectra for both, molecular azupyrene and ideal graphene, show asymmetry to higher BEs in the lineshape, which has previously been assigned to shake-up excitation of bound electrons close to the Fermi energy, as discussed below.[23] Graphene on Cu is n-doped,[24, 25] while the molecular orbitals of azupyrene hybridise with the delocalised electronic states of the Cu substrate,[26] resulting in both systems having an electronic density of states around the Fermi energy. This electronic configuration allows gapless excitation of electrons from occupied to unoccupied states, such that the emitted photoelectron can lose kinetic energy to promote these excitations, resulting in an asymmetrical XPS lineshape.

4. XPS measurements excluding contaminants and indicating self-limiting growth

In addition to the high resolution C 1s SXPS data shown in the main manuscript, wide range overview spectra were also acquired, shown in Figures S12-S14. In these spectra no first or second row p-block contaminant (e.g. B, N, O, Si, F, Cl, Br or I) was observed. For some samples a small Sn or Sb contaminant (maximum coverage ~1 Sn atom per 100 surface Cu atoms, in comparison to the Cu 3s core level) was observed, due to outgassing of the Mo clips that held the sample in place. This contaminant was transient, i.e. different batches of the Mo clips either did or did not have such a contamination. No systematic differences were observed between samples that contained these trace Sn / Sb contaminants and those that did not. No other contaminants were observed in the as-grown samples. As can be observed from these wide range overview spectra in Figures S12-S14, a variety of coverages were obtained as part of this study, roughly the coverage correlated with the deposition time, approximately 1 hour of deposition resulted in a complete monolayer, approximately 30 minutes of deposition resulted in half a monolayer coverage. Throughout the temperature of the evaporant (azupyrene) was kept constant. We observed a self-limiting growth for the defective graphene. Figure S15 shows the SXPS, of both a wide range overview spectra (Figure S15a) and a C 1s core level (Figures S15b) after deposition at intermediate temperatures for 90 and 180 minutes. After a 90 minute deposition the coverage is close to that expected for a saturated carbon monolayer (comparing the C 1s to Cu 3p intensity suggests an atomic density of the C atoms of ~40 atoms/nm^2. NB. that this estimate is based on the overview spectra shown in Figure S15a and will therefore have a large associated uncertainty). Doubling the growth time to 180 minutes resulted only a very small

(< 1%) increase in the area of the C 1s signal. Furthermore, post annealing of the film in vacuum to elevated temperatures where graphene growth, under an azupyrene flux, is observed, resulted in no apparent change to the C 1s signal. This lack of change indicates that the structure of the network is determined by the temperature during growth and that the grown defective films are thermally stable up to elevated temperatures. Bilayers and trilayers of graphene result in a measurable shift to higher binding energy[27], no such species was observed in this study excluding the presence of any appreciable bi- or tri-layer.

Figures S12-S14

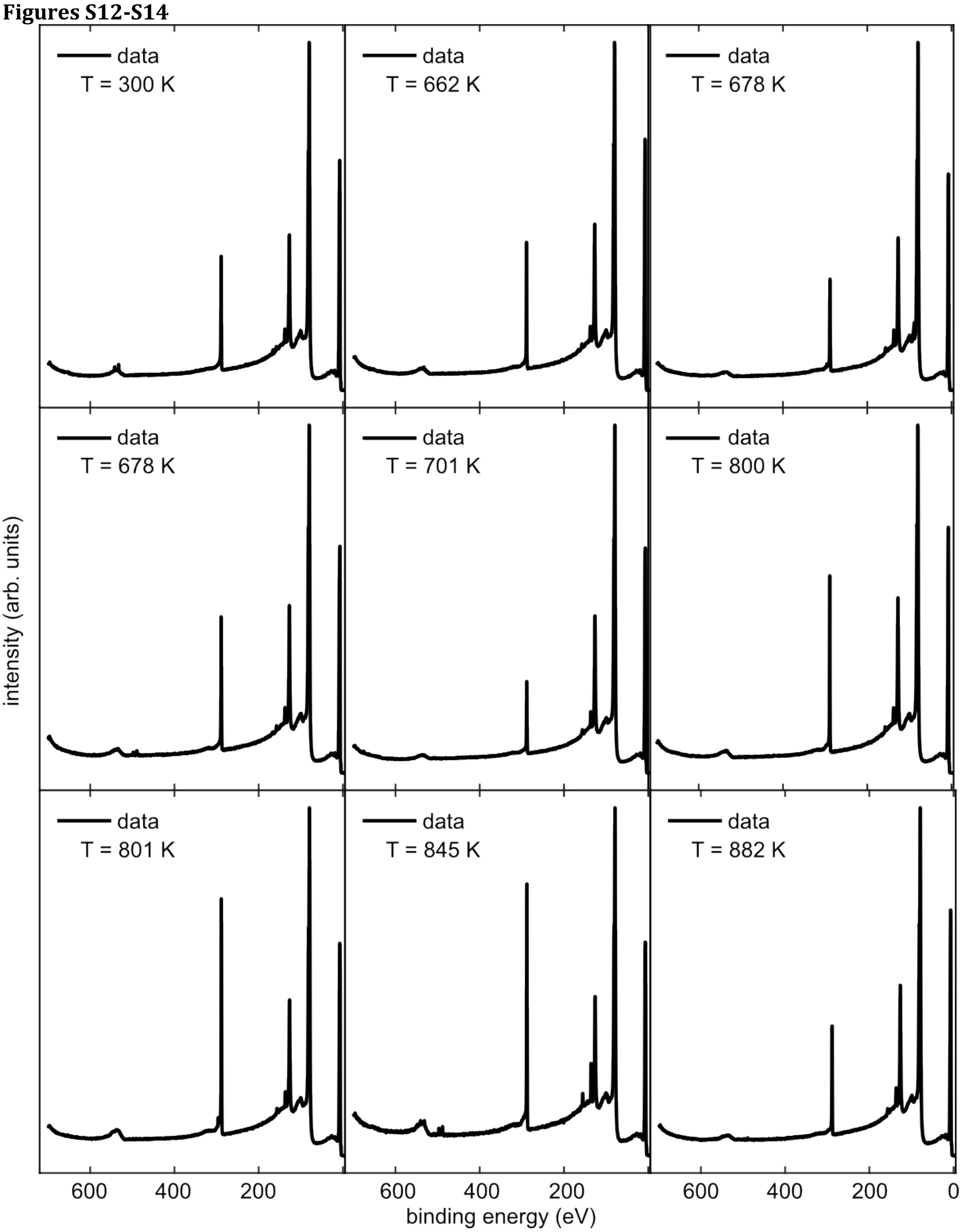

Figure S12: Overview SXPS data taken from the samples for which the NIXSW data was performed (see Figure 4b,c in main manuscript). Indicated is the estimated substrate growth temperature for each sample. All spectra were obtained at a photon energy of 850 eV and the binding energy is uncorrected. Sharp features at ~450 eV correspond to Sn contamination, ~550 eV to Sb contamination. The broad feature at ~550 eV is the C KLL Auger decay spectra. Features at 136 and 156 eV binding energy are the Cu 2p spectra originating from the second order light from the soft X-ray monochromator ($h\nu = 1700$ eV).

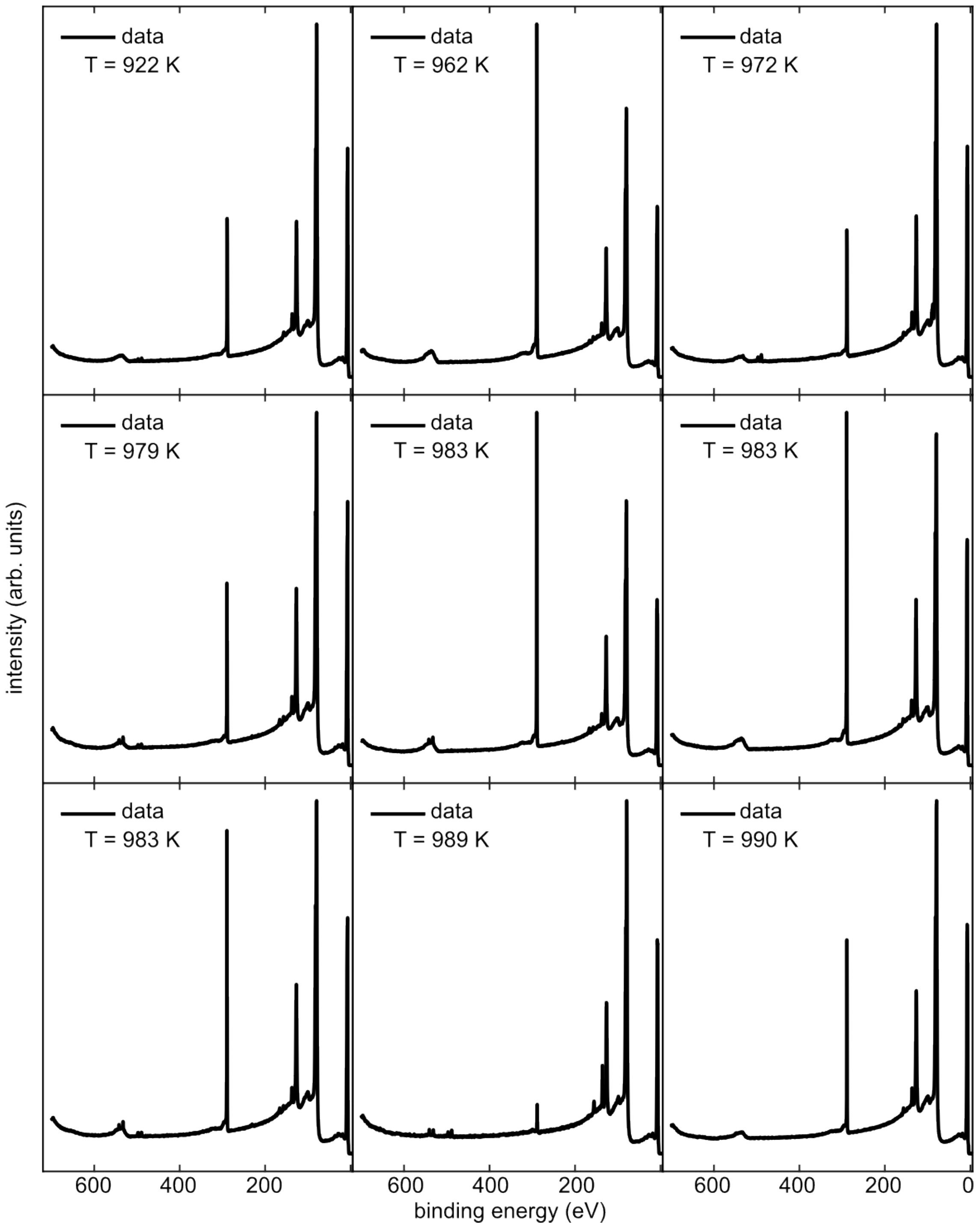

Figure S13: Overview SXPS data taken from the samples for which the NIXSW data was performed (see Figure 4b,c in main manuscript). Indicated is the estimated substrate growth temperature for each sample. All spectra were obtained at a photon energy of 850 eV and the binding energy is uncorrected. Sharp features at ~450 eV correspond to Sn contamination, ~550 eV to Sb contamination. The broad feature at ~550 eV is the C KLL Auger decay spectra. Features at 136 and 156 eV binding energy are the Cu 2p spectra originating from the second order light from the soft X-ray monochromator ($h\nu = 1700$ eV).

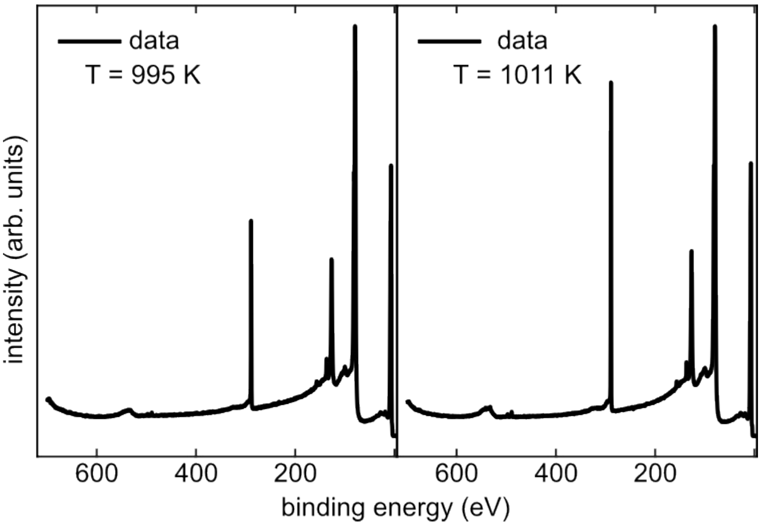

Figure S14: Overview SXPS data taken from the samples for which the NIXSW data was performed (see Figure 4b,c in main manuscript). Indicated is the estimated substrate growth temperature for each sample. All spectra were obtained at a photon energy of 850 eV and the binding energy is uncorrected. Sharp features at ~450 eV correspond to Sn contamination, ~550 eV to Sb contamination. The broad feature at ~550 eV is the C KLL Auger decay spectra. Features at 136 and 156 eV binding energy are the Cu 2p spectra originating from the second order light from the soft X-ray monochromator ($h\nu = 1700$ eV).

Figure S15

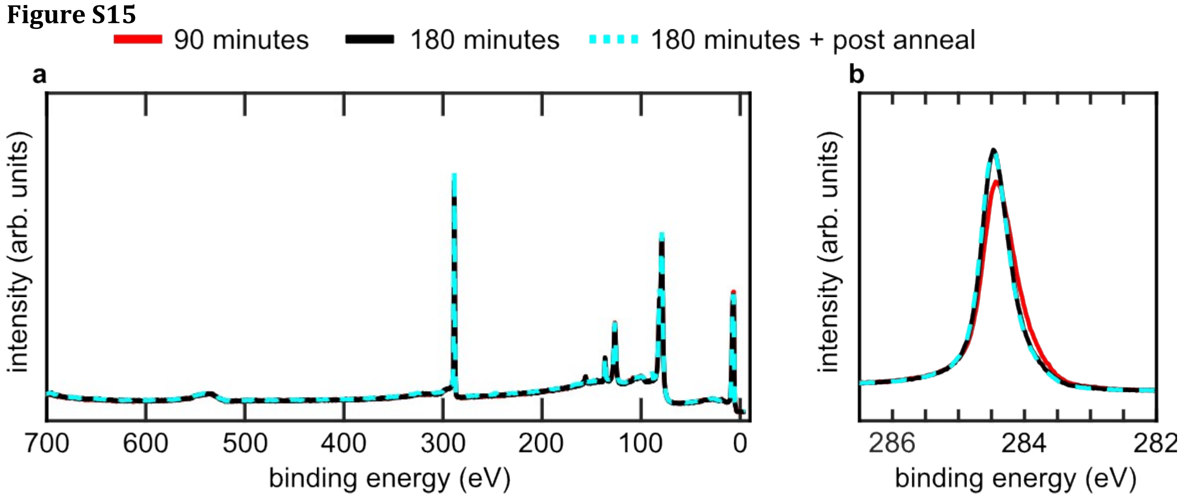

Figure S15: (**a**) Overview and (**b**) C 1s SXPS data from defective graphene films grown for protracted deposition times (90, red line, and 180 minutes, black line), indicating that the film is self-limited to a single monolayer at high coverage. Also shown is the result of post annealing (dashed, cyan line) a defective graphene film in vacuum, to temperatures where the growth of graphene under an azupyrene flux is observed, resulting in no observable changes in the spectra.

5. Further discussion on the experimental NEXAFS data

The near-edge X-ray absorption fine structure (NEXAFS) spectroscopy obtained from molecular azupyrene and graphene grown on Cu(111) are shown in Figure 3d of the main manuscript. In both the π^* region (Figure 3f) and the σ^* region (Figure 3g) clear differences are present between molecular azupyrene and graphene. In the π^* region, the first peak of molecular azupyrene occurs ~0.7 eV lower in photon energy than graphene and, in the σ^* region, instead of a sharp resonant feature, a broad one is observed instead for azupyrene.

6. Details on the computed XPS and NEXAFS data: Model structures

Surface slab models of metal-adsorbed graphene were built based on previously moiré superstructure of a $3\sqrt{13} \times 3\sqrt{13}$ Cu(111) surface slab that accommodates a $2\sqrt{31} \times 2\sqrt{31}$ graphene layer with an angle of rotation of 5°.[16] The total number of carbon atoms is 248. The total number of copper atoms is 936. Structures were relaxed with the all-electron code FHI-aims[11] and the PBE+MBD-NL functional[28] with other settings reported in detail in Ref. [16]. Surface slab models were optimized with eight metal layers where the bottom six layers were frozen in their bulk truncated structure. Using this approach, we created relaxed structures for a free-standing ideal graphene layer, a free-standing Stone-Wales defective graphene layer,

ideal graphene adsorbed on Cu(111) and a graphene layer with a Stone-Wales defect centred atop a bridge site. The corresponding structures are shown in Figure S16. For the subsequent XPS simulations, the number of metal layers was reduced to four. For the NEXAFS simulations, the number of metal layers was reduced to two.

7. Details on the computed XPS and NEXAFS data: XPS calculations

XPS and NEXAFS C 1s spectra were simulated with core hole calculations based on the optimized structures with the CASTEP code, version 21.1[12] using the PBE exchange-correlation functional[13]. In each calculation, a core-hole-excited pseudopotential was created for the target atom and the calculation was repeated for each targeted atom. We closely follow the procedure explained in detail in Ref. [19]. XPS simulations were performed with the Delta-Self-Consistent-Field (ΔSCF) method. Here, for each carbon atom an excited core-hole calculation was performed, where the electron configuration of the respective pseudopotential was modified to [$1s^1$, $2s^2$, $2p^3$] to localise the core-hole. The additional valence electron was removed, and the net positive charge was compensated by a homogeneous background charge of −1.0 e to achieve net neutrality in the unit cell. XP spectra were broadened with a pseudo-Voigt function to capture instrumental and lifetime broadening effects.[20] For the SXPS modelling, a Gaussian full width half maximum (FWHM) of 0.40 eV and a Lorentzian FWHM of 0.46 eV were used. A global shift on the simulated spectrum is applied to align with experimental binding energies. For the Stone-Wales defect structure reported in Figure 3 of the main manuscript, XPS binding energies were calculated for all carbon atoms within the Stone-Wales defect and in the 1st, 2nd, 3rd, and 4th neighbour shell. XPS binding energies were calculated for 16 randomly placed carbon atoms for the metal-adsorbed ideal graphene sheet. The atoms considered in the XPS and NEXAFS simulations are indicated in colour in the Figure S16.

8. Details on the computed XPS and NEXAFS data: NEXAFS calculations

Simulations were carried out using the ELNES module[29] in CASTEP. A half core-hole was introduced into the pseudopotential for carbon by changing the electron configuration of the pseudopotential to [$1s^{1.5}$, $2s^2$, $2p^{2.5}$]. Each core-hole calculation requires a self-consistent total energy calculation followed by a band structure and dipole matrix element calculation to converge the unoccupied states. 1400, 900, and 2000 unoccupied states were considered, for the SW defect, the metal-adsorbed graphene and the free-standing ideal graphene, which was the limit achievable with the computational memory and time constraints. The total NEXAFS spectrum is produced by summing all single carbon species contributions and shifting them with the previously calculated XPS core binding energy. An energy-dependent broadening scheme was applied to account for the reduced lifetime at increasing excitation energies. The NEXAFS spectrum is divided into three ranges. The first range starts with the leading edge, spans the first 5 eV of the spectrum and is assigned a pseudo-Voigt FWHM of 0.75 eV and a 80%/20% Gaussian/Lorentzian ratio, while the third range starts from

15 eV above the leading edge and is assigned a FWHM of 2.0 eV and a 20%/80% G/L ratio. Both ranges are connected by an intermediate range, in which the FWHM and the G/L ratio change linearly. A global energy shift is applied to the final broadened NEXAFS spectrum to align with the experimental data. Due to the large computational cost of the NEXAFS spectra, only a subset of the atoms for which XP binding energies were calculated were considered in the NEXAFS simulations. Note the simulated spectra only consider contributions from atoms within the defect and should only be considered indicative of the source of features in the experimental spectra.

Figure S16

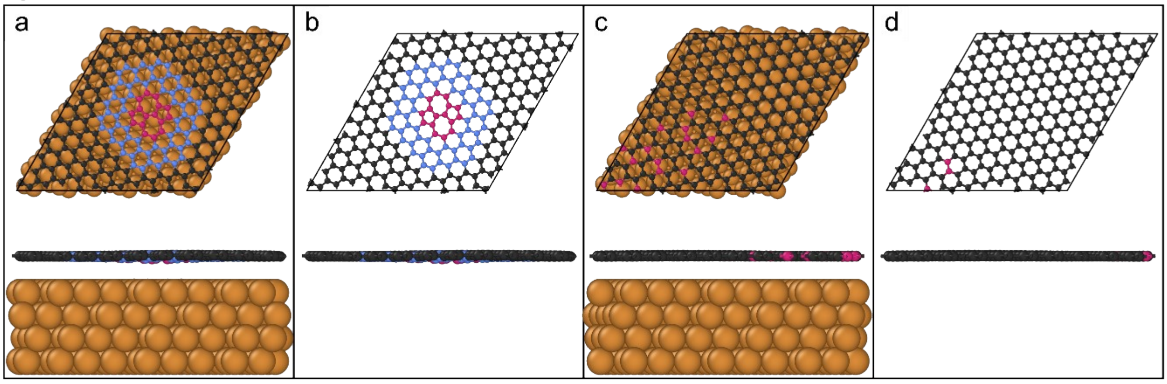

Figure S16: Top and side views of the calculated moiré pattern of graphene with a Stone-Wales defect (**a**) adsorbed on a Cu(111) surface and (**b**) freestanding. Top and side views of the calculated moiré pattern of ideal graphene (**c**) adsorbed on a Cu(111) surface

and (**d**) freestanding. Carbon atoms colored red were used in the XPS and NEXAFS calculations, colored blue in the XPS calculations only and colored black were not used in either spectroscopy calculation. Note that the NEXAFS calculations were performed with the bottom 2 Cu layers removed (not depicted) to maintain tractability with the increased computational cost of performing NEXAFS simulations. Visualisations were created with Ovito.[30]

9. Shifting of the binding energy and photon energy scales in the calculated spectra

In Figure 3, in the main article, the simulated spectra in panel **b** have been shifted so that the maximum intensity in the spectrum is the same as that measured experimentally for the SXPS data of the defective film. In panels **e,h** and **i** have been shifted in photon energy so that the graphene π^* resonance matches that of the experimental data and have been rescaled in intensity such that the minima between the π^* and σ^* region overlaps for the defective and graphene theoretical data. The simulated XPS and NEXAFS spectra without an applied shift in binding / photon energy are shown in Figure S17. NB. in Figure 3**g** the spectra have been offset such that all spectra have identical intensity at a photon energy of 289 eV.

Figure S17

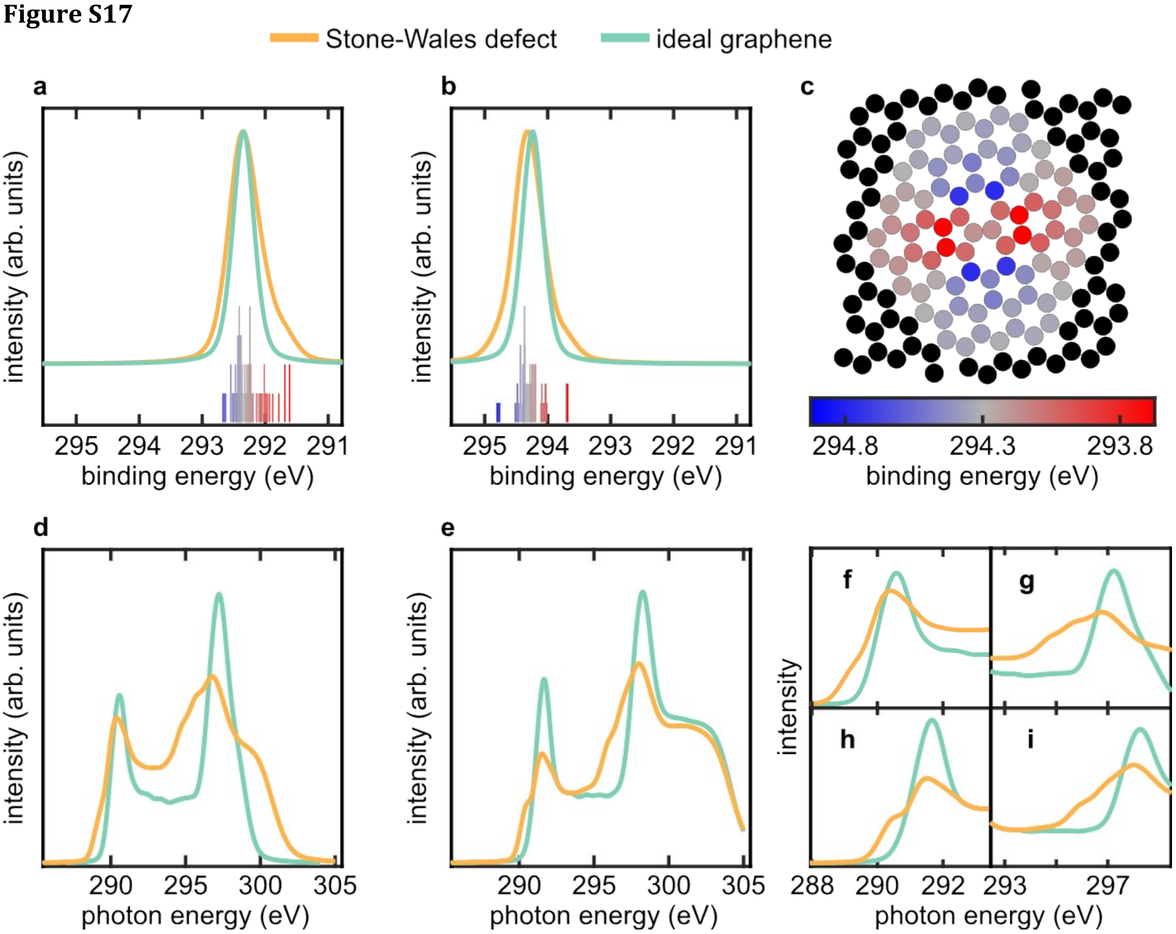

Figure S17: Computed XPS of a Stone-Wales defect embedded in a graphene mesh and the associated ideal graphene mesh (**a**) on Cu(111) and (**b**) free-standing, with a histogram of the theoretical binding energies of the Stone-Wales defect spectrum, without broadening, using the same color scale as shown in panel (**c**). Also shown (**c**) is a top view depiction of the structure employed in the free-standing DFT calculations for the Stone-Wales defect, showing the binding energy of each calculated atom as per the indicated color scale. Computed NEXAFS of a Stone-Wales defect embedded in a graphene mesh and the associated ideal graphene mesh (**d,f,g**) on Cu(111) and (e,h,i) free-standing. Note that these spectra are not offset to match the experimental data, but are shown with their calculated photon and binding energies.

10. Normal incidence X-ray standing waves

The normal incidence X-ray standing wave (NIXSW) technique[31] utilises the X-ray standing wave formed by the constructive / deconstructive interference between the incident and reflected waves close to the Bragg condition for a given Bragg reflection H=(h,k,l). The period of this standing wave matches that of the interplanar spacing d_H between the Bragg diffraction planes[32]. The phase of the standing wave varies when the photon energy is scanned through the Bragg condition. This phase change results in the location of the maximum intensity of the standing wave with respect to the Bragg diffraction planes itself varying. When the phase is zero, the maximum intensity of the standing wave lies halfway between Bragg diffraction planes; when the phase is π the maximum intensity is coincident with the Bragg diffraction planes. In the

case of face centred cubic crystal made up of a single element, the Bragg diffraction planes will be coincident with the real space atomic planes of the material. Any atom immersed in this standing wavefield will experience a varying electromagnetic field intensity as a function of its position between these diffraction planes. This varying field intensity will result in characteristic absorption profiles that can be monitored by the photoelectron intensity profile. The measured profile is then fitted uniquely, using dynamical diffraction theory,[33] by two dimensionless parameters[31]: the coherent fraction, f_H, and the coherent position, p_H. These, respectively, broadly correspond to the degree of order and the mean position of the absorber atoms relative to the Bragg diffraction planes. When the chosen Bragg diffraction plane is parallel with the surface plane the coherent position is related to the mean adsorption height of a species by:

$$h_H = (n + p_H) \cdot d_H, \quad (1)$$

where n is an integer and relates to so called "modulo-d" ambiguity[34]. This ambiguity means that adsorption heights that differ by the interplanar spacing cannot be directly differentiated. However, in practice the correct value of n can often be easily assigned as d_H typically is in the order of ~2 Å, thus it is generally trivial to exclude adsorption heights that are unphysically low or high.

To model the experimentally measured coherent fraction and coherent position one must sum over all positions that the measured atoms take within the layer spacing defined by the Bragg diffraction plane. As such:

$$f_H \cdot \exp(i \cdot 2\pi p_H) = \sum_{n=1}^{n=m} f_n \cdot \exp(i \cdot 2\pi p_n), \quad (2)$$

where f_n is the fraction of atoms that are at the coherent position, p_n and $n = 1 \rightarrow m$ are all the possible positions that the species can take. If one considers a simple case, with an equal mixture of two adsorption sites, equation (2) becomes:

$$f_H \cdot \exp(i \cdot 2\pi p_H) = \frac{\exp(i \cdot 2\pi p_1)}{2} + \frac{\exp(i \cdot 2\pi p_2)}{2}. \quad (3)$$

For the special case where p_1 differs from p_2 by 0.5 then,

$$f_H \cdot \exp(i \cdot 2\pi p_H) = \frac{\exp(i \cdot 2\pi p_1)}{2} + \frac{\exp(i \cdot 2\pi (p_1 + 0.5))}{2}, \quad (4)$$

$$f_H \cdot \exp(i \cdot 2\pi p_H) = \frac{\exp(i \cdot 2\pi p_1)}{2} - \frac{\exp(i \cdot 2\pi p_1)}{2}, \quad (5)$$

$$f_H \cdot \exp(i \cdot 2\pi p_H) = 0, \quad (6)$$

$$f_H = 0. \quad (7)$$

Thus, if a species constitutes two primary adsorption heights that happen to differ by approximately half of the d-spacing related to the measured Bragg plane, then the expected measured coherent fraction would be very low.

11. Calibration of sample growth temperature

The various films presented in this work were grown in different sample preparation chambers, specifically an offline growth chamber at Diamond Light Source, the growth chamber directly mounted on the I09 beam line at Diamond Light Source, the growth chamber directly mounted on the FlexPES beam line at MAX IV and the growth chamber directly mounted on the nc-AFM system at the Technical University of Munich. On the two growth chambers at Diamond Light Source there was no thermocouple mounted close to the sample and, through the many sample preparations presented in this work, the methodology for mounting the samples onto the sample plates differed significantly. In short, there is significant uncertainty in the temperatures used for the growth of the samples at Diamond Light Source. However, the samples grown in the growth chamber directly mounted on the FlexPES beam line at MAX IV had a thermocouple directly mounted at the sample. Excluding all nc-AFM samples (Figure 3) and STM measurements of the molecular and graphene films shown in Figure 1 of the main article, every single sample presented in this work was

measured by SXPS, thus by comparison of the SXPS data for the samples measured on the FlexPES beam line at MAX IV to the SXPS data measured for all other samples, we can obtain an estimate of the sample growth temperature. Specifically, as shown in Figure S18, the MAX IV SXPS data shows a clear linear variation in the binding energy of the maximum in intensity as a function of growth temperature. By fitting the linear variation observed in the MAX IV data we could estimate the growth temperature of the samples grown at Diamond Light Source by using the binding energy of the maximum intensity measured in their respective SXP spectra.

Figure S18

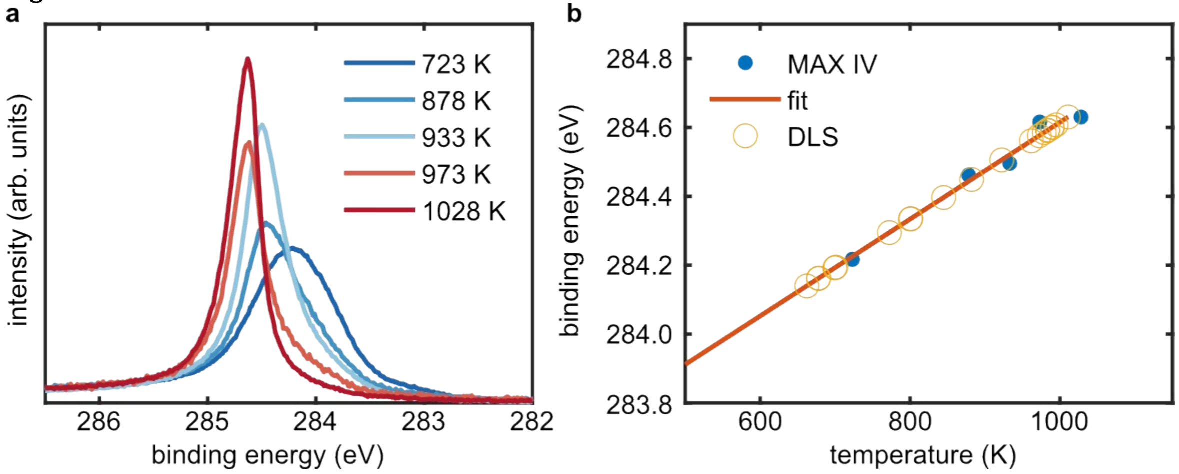

Figure S18: (**a**) XPS obtained of the samples grown in the sample growth chamber mounted directly onto the FlexPES beam line at MAX IV and (**b**) the binding energy of the maximum intensity plotted against their growth temperature that was measured directly by a thermocouple mounted onto the sample. The linear trend of the binding energy of the maximum intensity as a function of growth temperature is fitted with a straight line and used to estimate the growth temperature of the samples grown at Diamond Light Source (DLS).

Figures S19-S25

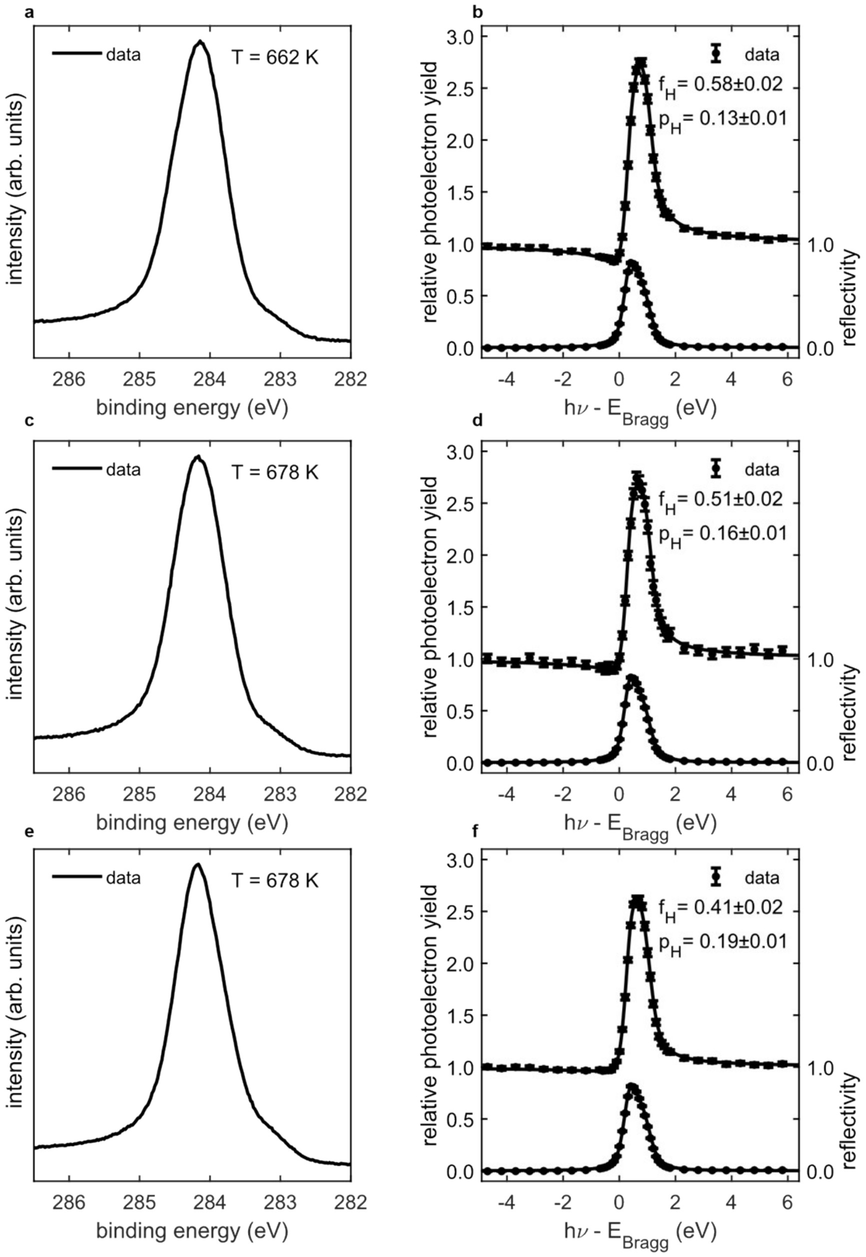

Figure S19: (**a**,**c**,**e**) Experimental C 1s SXPS and (**b**,**d**,**f**) NIXSW data for films grown on Cu(111) held at the indicated estimated temperature under exposure to azupyrene. The indicated coherent fractions and coherent positions, obtained from fitting the NIXSW form the data shown in Figure 4b,c in the main manuscript.

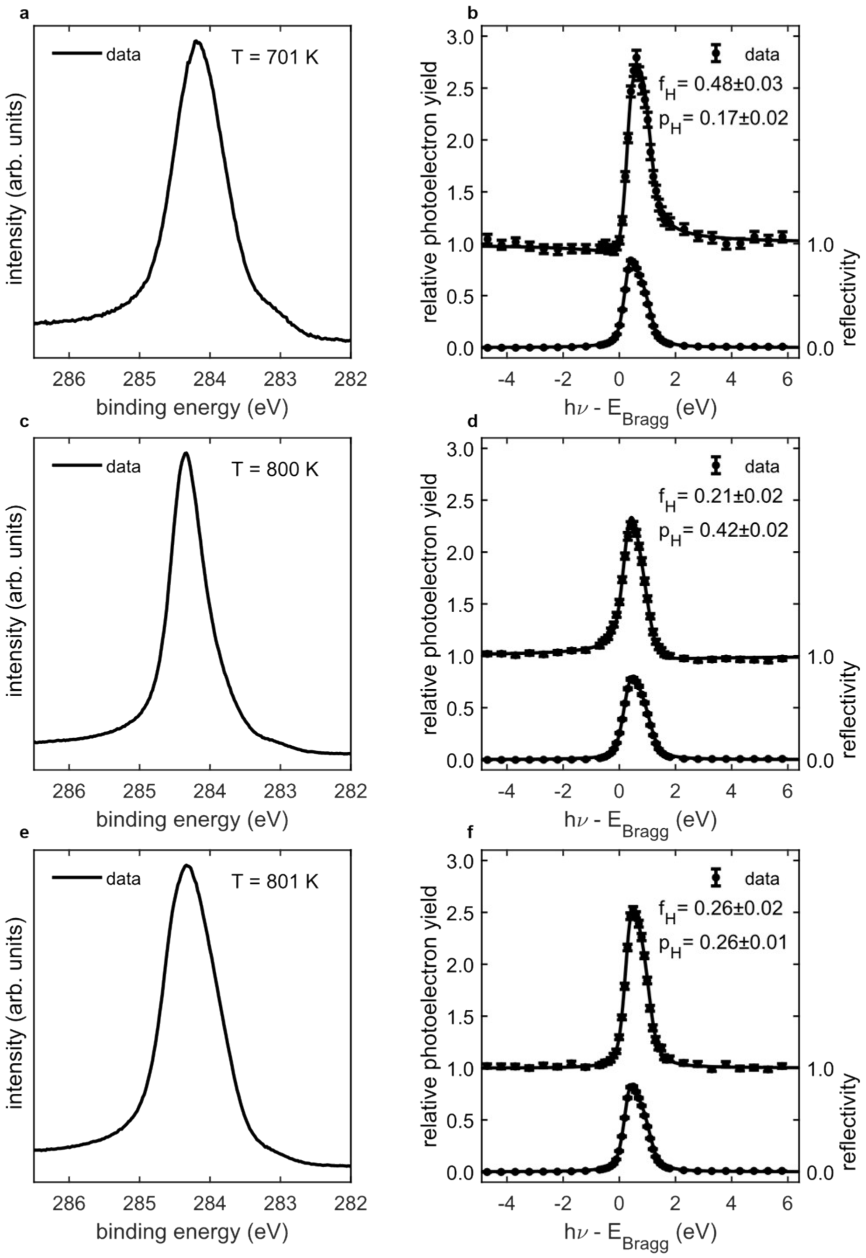

Figure S20: (**a**,**c**,**e**) Experimental C 1s SXPS and (**b**,**d**,**f**) NIXSW data for films grown on Cu(111) held at the indicated estimated temperature under exposure to azupyrene. The indicated coherent fractions and coherent positions, obtained from fitting the NIXSW form the data shown in Figure 4b,c in the main manuscript.

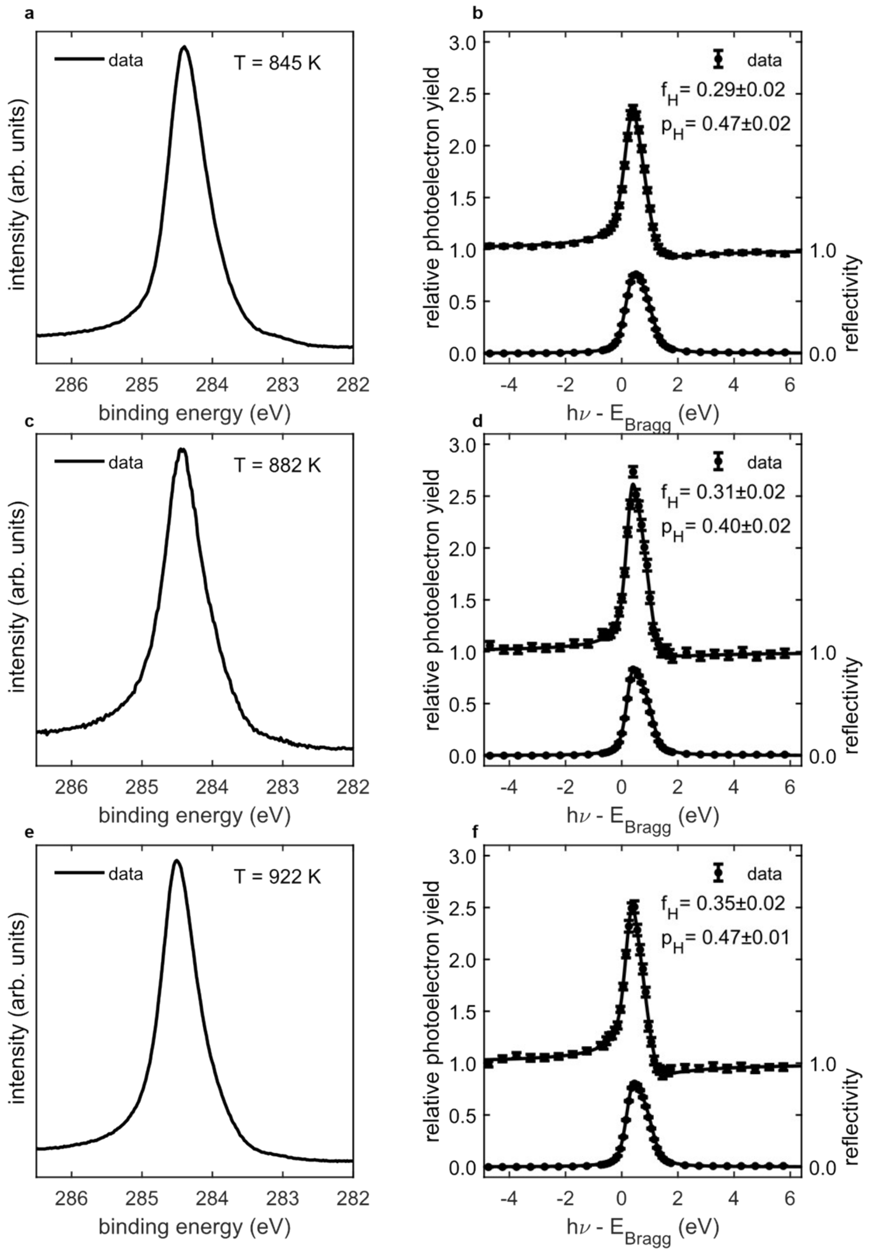

Figure S21: (**a**,**c**,**e**) Experimental C 1s SXPS and (**b**,**d**,**f**) NIXSW data for films grown on Cu(111) held at the indicated estimated temperature under exposure to azupyrene. The indicated coherent fractions and coherent positions, obtained from fitting the NIXSW form the data shown in Figure 4b,c in the main manuscript.

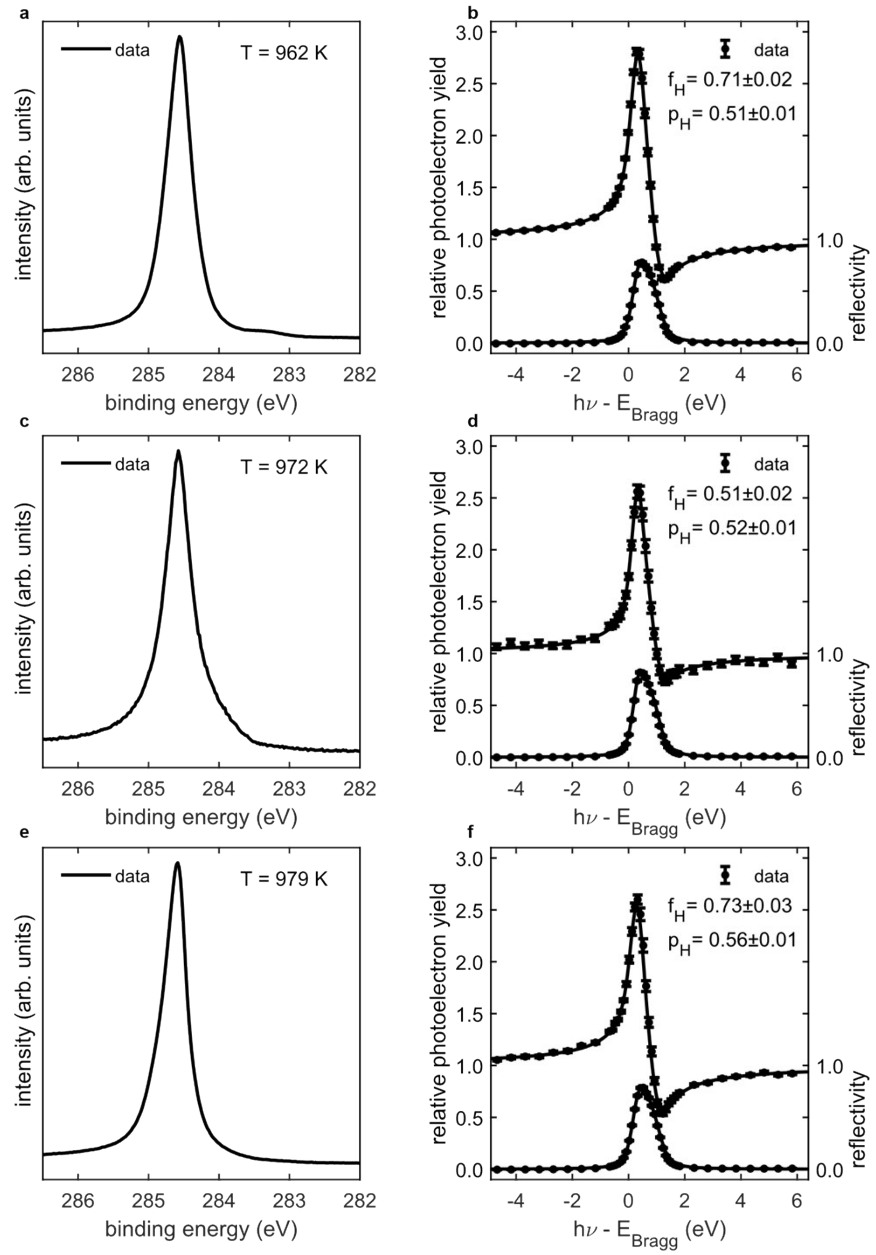

Figure S22: (**a**,**c**,**e**) Experimental C 1s SXPS and (**b**,**d**,**f**) NIXSW data for films grown on Cu(111) held at the indicated estimated temperature under exposure to azupyrene. The indicated coherent fractions and coherent positions, obtained from fitting the NIXSW form the data shown in Figure 4b,c in the main manuscript.

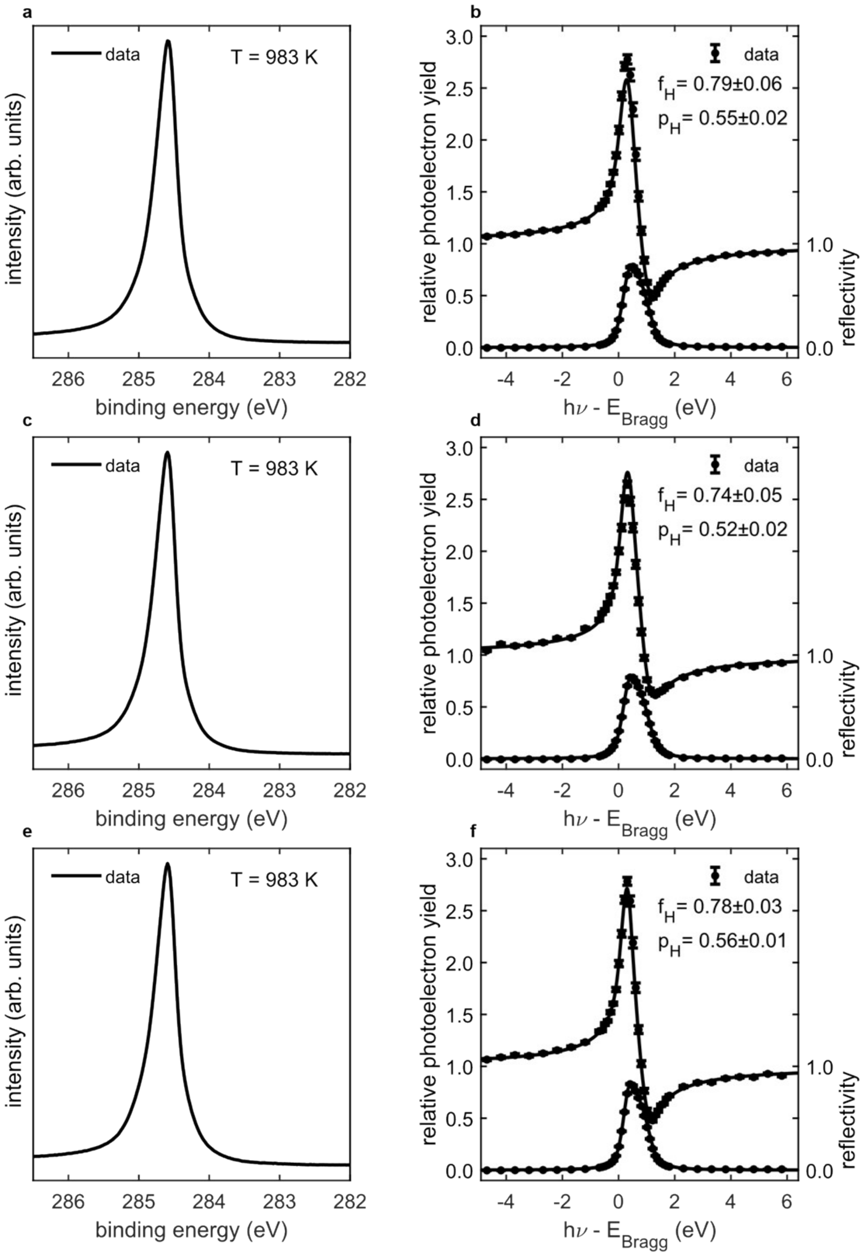

Figure S23: (**a**,**c**,**e**) Experimental C 1s SXPS and (**b**,**d**,**f**) NIXSW data for films grown on Cu(111) held at the indicated estimated temperature under exposure to azupyrene. The indicated coherent fractions and coherent positions, obtained from fitting the NIXSW form the data shown in Figure 4b,c in the main manuscript.

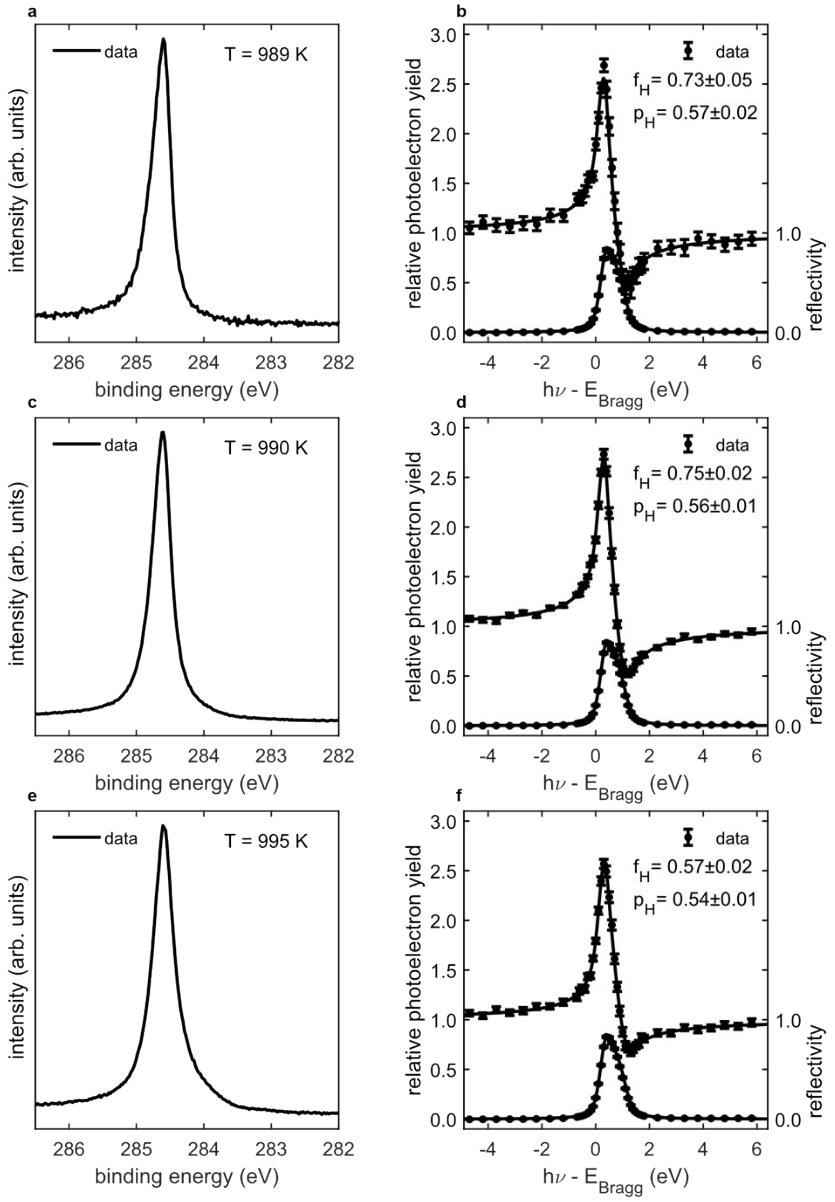

Figure S24: (**a**) Experimental C 1s SXPS and (**b**) NIXSW data for films grown on Cu(111) held at the indicated estimated temperature under exposure to azupyrene. The indicated coherent fractions and coherent positions, obtained from fitting the NIXSW form the data shown in Figure 4b,c in the main manuscript.

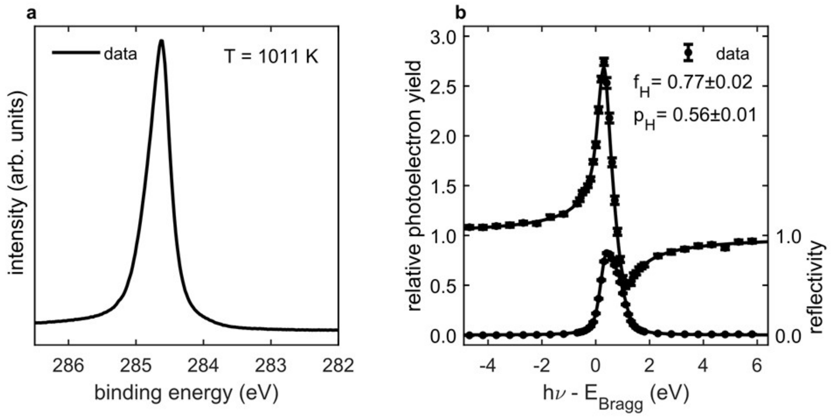

Figure S25: (a,c,e) Experimental C 1s SXPS and (b,d,f) NIXSW data for films grown on Cu(111) held at the indicated estimated temperature under exposure to azupyrene. The indicated coherent fractions and coherent positions, obtained from fitting the NIXSW form the data shown in Figure 4b,c in the main manuscript.

12. Further details on the ADF-STEM measurements

The enumerated frequency of the identified 4- to 8-membered rings can be found in Table S1. Small bright protrusions were observed on occasion, e.g. the circled areas in Figure S22, which may indicate small clusters of large Z atoms that have remained on defect sites. These protrusions are very small, potentially even single atoms. It is well established that the high energy electron beam used in TEM measurement can induce 5- and 7-membered defects, such as those observed in this study, though it is more likely to heal such a defect than create one.[35, 36] As such, we performed the measurements in a low energy mode with a reduced electron flux to reduce the probability of defect introduction. In this regime, the probabilistic rate of beam induced 5-/7-membered ring formation would yield one ring per scan. Considering the rate of C ejection observed by Meyer et al.[37], the expected defect rate induced by the beam in our AC-STEM measurements would be ~1 defect per image.

Figure S26

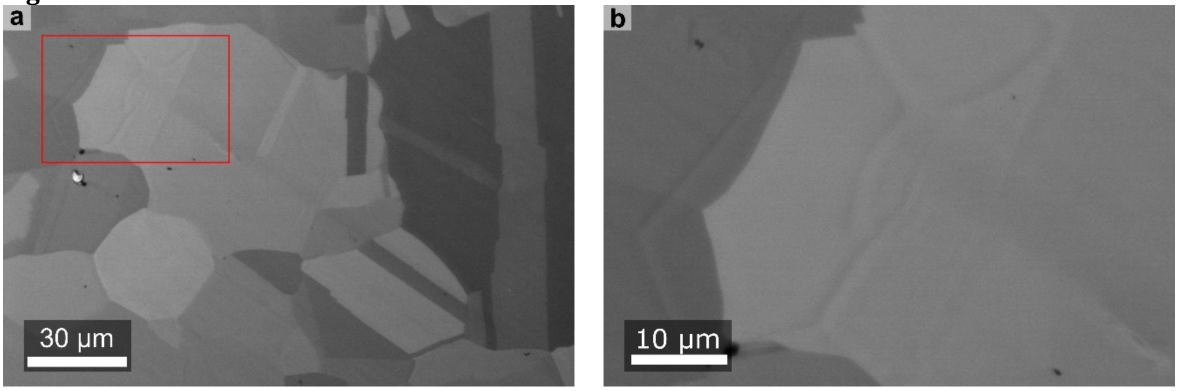

Figure S26: **(a,b)** Scanning electron microscopy (SEM) measurements of the defective film grown on Cu foil. In the SEM measurement several domains of the Cu foil can be clearly observed, in the zoomed in region **(b)**, taken from the region highlighted by the red box in panel **(a)**, one can also see the domains of the monolayer defective graphene contrasted against the domains on the Cu foil. The defective film appeared to cover the whole surface, without any indication of bilayer formation.

Figure S27

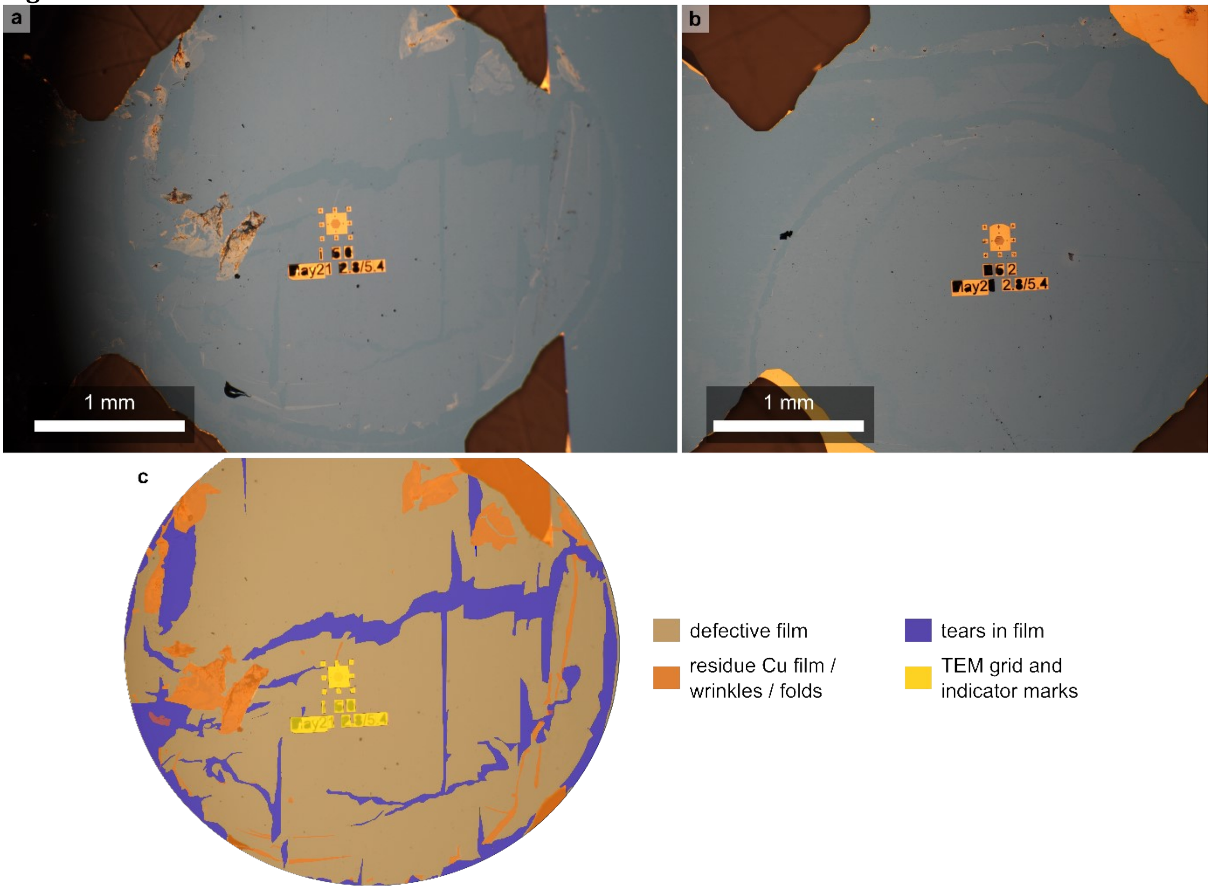

Figure S27: **(a,b)** Optical microscopy of the sample grown on Cu foil for TEM measurements, post-etching of the growth substrate and removal of the protective PMMA support. The two images were taken from two different segments of the grown film, separated by several millimetres. **(c)** For clarity, regions of panel **(a)** are overlayed with a colour scheme to highlight different details. While there are some observable tears in the film from the transfer process (blue), and some residual Cu films, wrinkles and folds present (orange) it is notable that the defective film (yellow) is shown to cover a large area of the image with clear continuous films on the millimetre scale. Note: as described in the methods, §1.6, the foil was coated in a tape with holes in the centre – roughly the position of these holes align with the large amount of residual Cu at the edges of the image in **(a,b).**

Figures S28-32

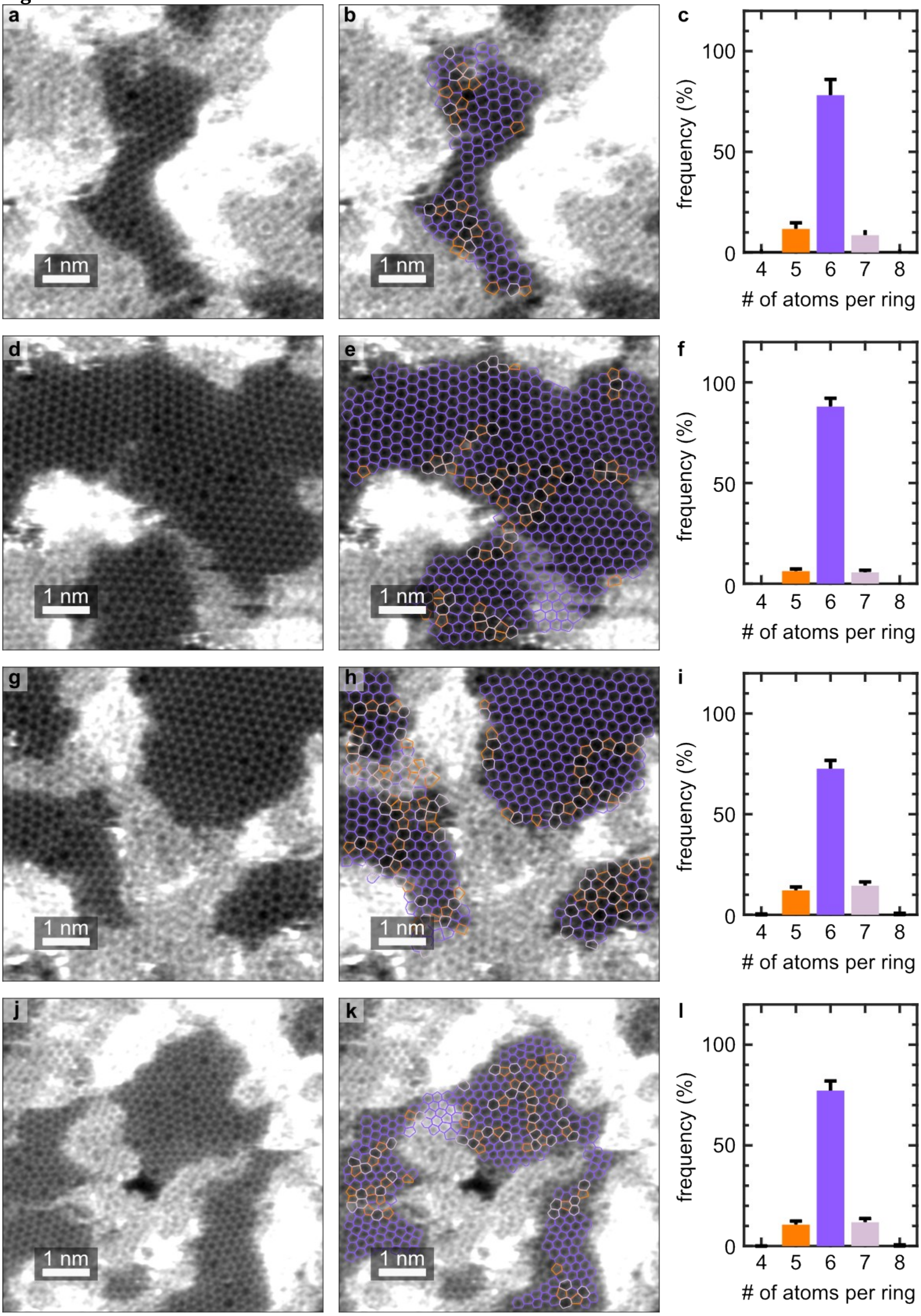

Figure S28: (**a**-**b**,**d**-**e**,**g**-**h**,**j**-**k**) Atomically resolved AC-STEM measurements of a defective film. Overlayed in panels (**b**,**e**,**h**,**k**) are the C-C bonds, 5-membered rings are dark orange, 7-membered are light purple and 6-membered are dark purple. Also shown in panels (**c**,**f**,**i**,**l**) are the histograms of the relative frequency of 4- to 8-membered rings.

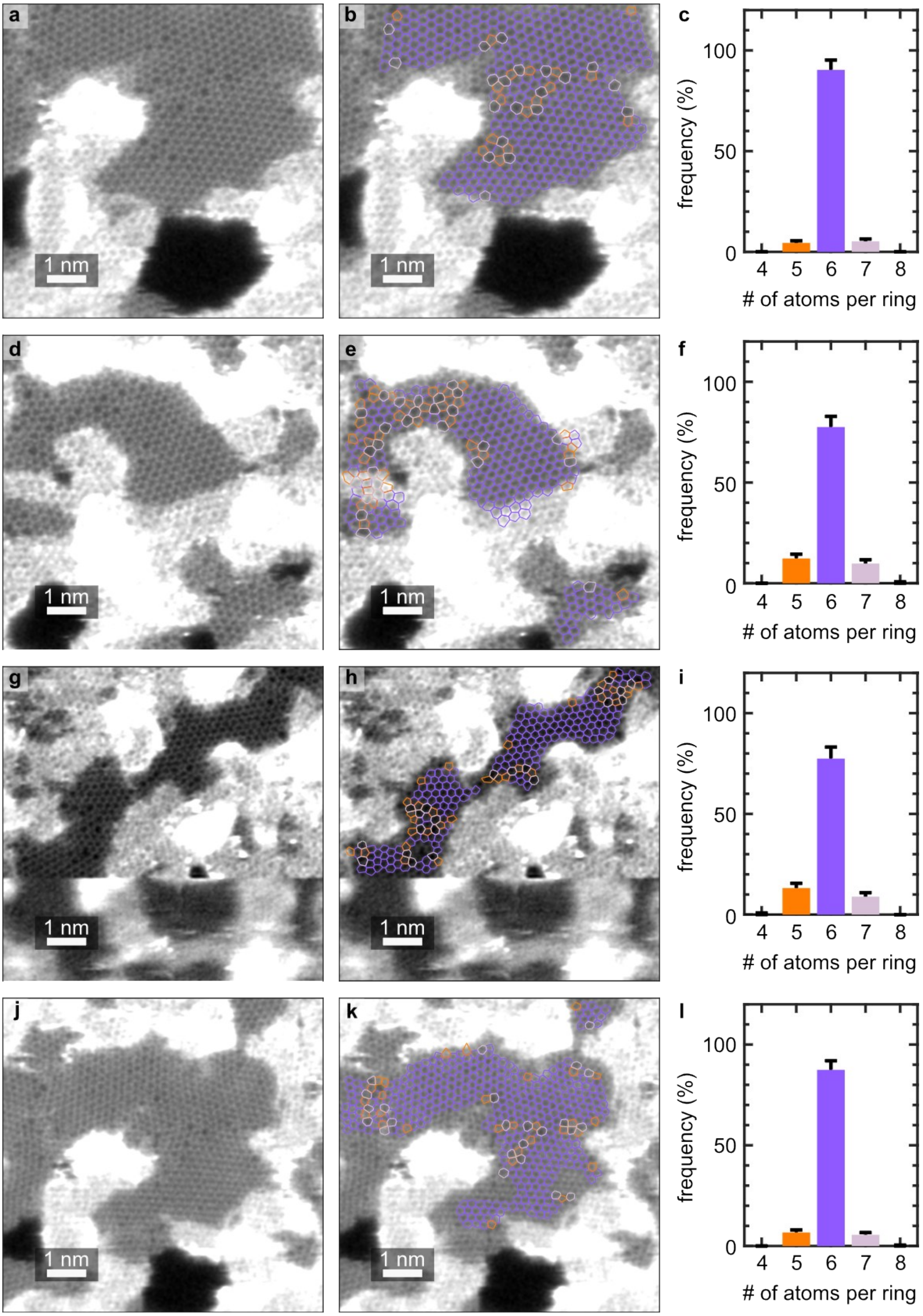

Figure S29: (**a**-**b**,**d**-**e**,**g**-**h**,**j**-**k**) Atomically resolved AC-STEM measurements of a defective film. Overlayed in panels (**b**,**e**,**h**,**k**) are the C-C bonds, 5-membered rings are dark orange, 7-membered are light purple and 6-membered are dark purple. Also shown in panels (**c**,**f**,**i**,**l**) are the histograms of the relative frequency of 4- to 8-membered rings.

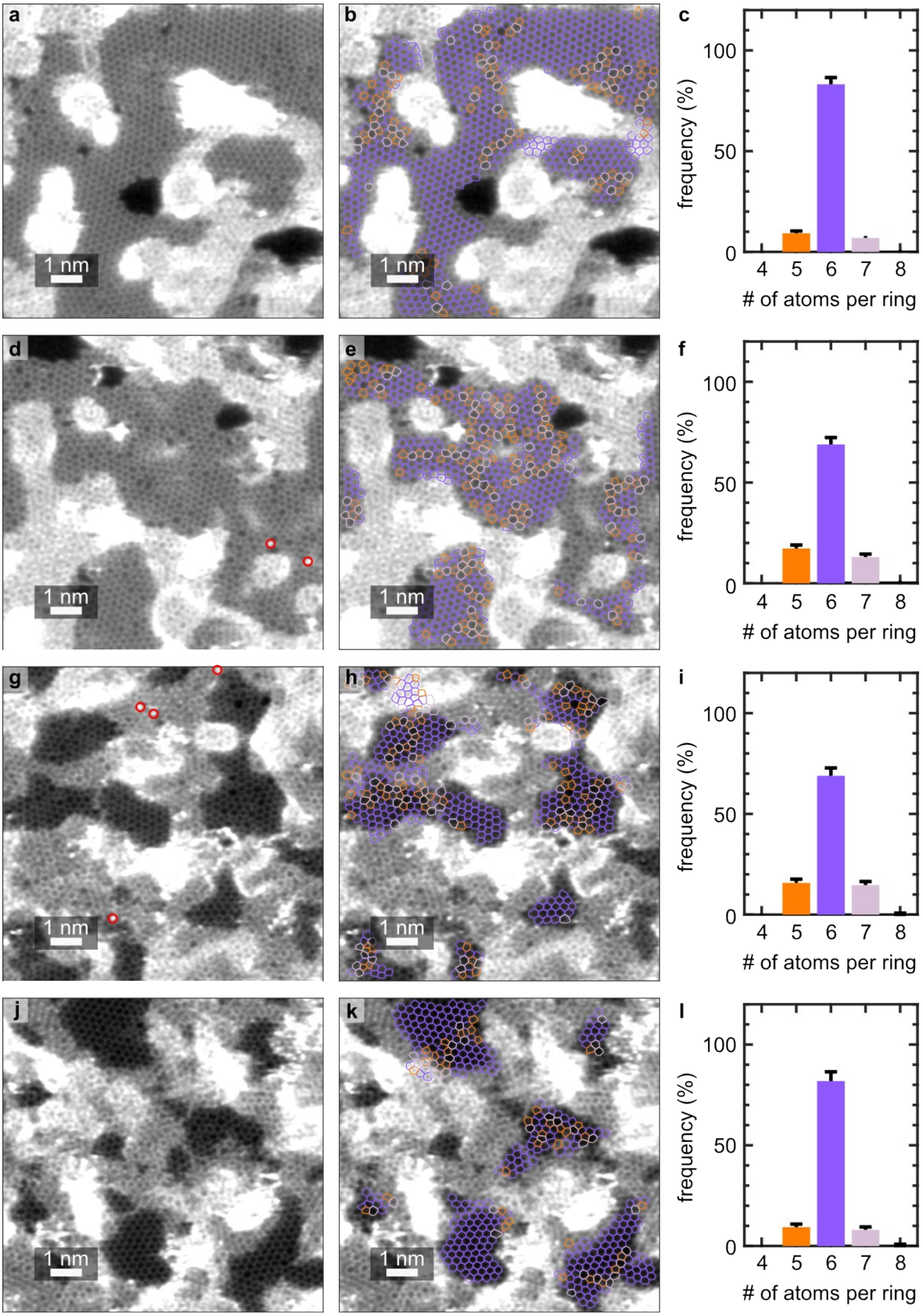

Figure S30: (**a**-**b**,**d**-**e**,**g**-**h**,**j**-**k**) Atomically resolved AC-STEM measurements of a defective film. Red circles highlight protrusions, that may relate to single metal atoms. Overlayed in panels (**b**,**e**,**h**,**k**) are the C-C bonds, 5-membered rings are dark orange, 7-membered are light purple and 6-membered are dark purple. Also shown in panels (**c**,**f**,**i**,**l**) are the histograms of the relative frequency of 4- to 8-membered rings.

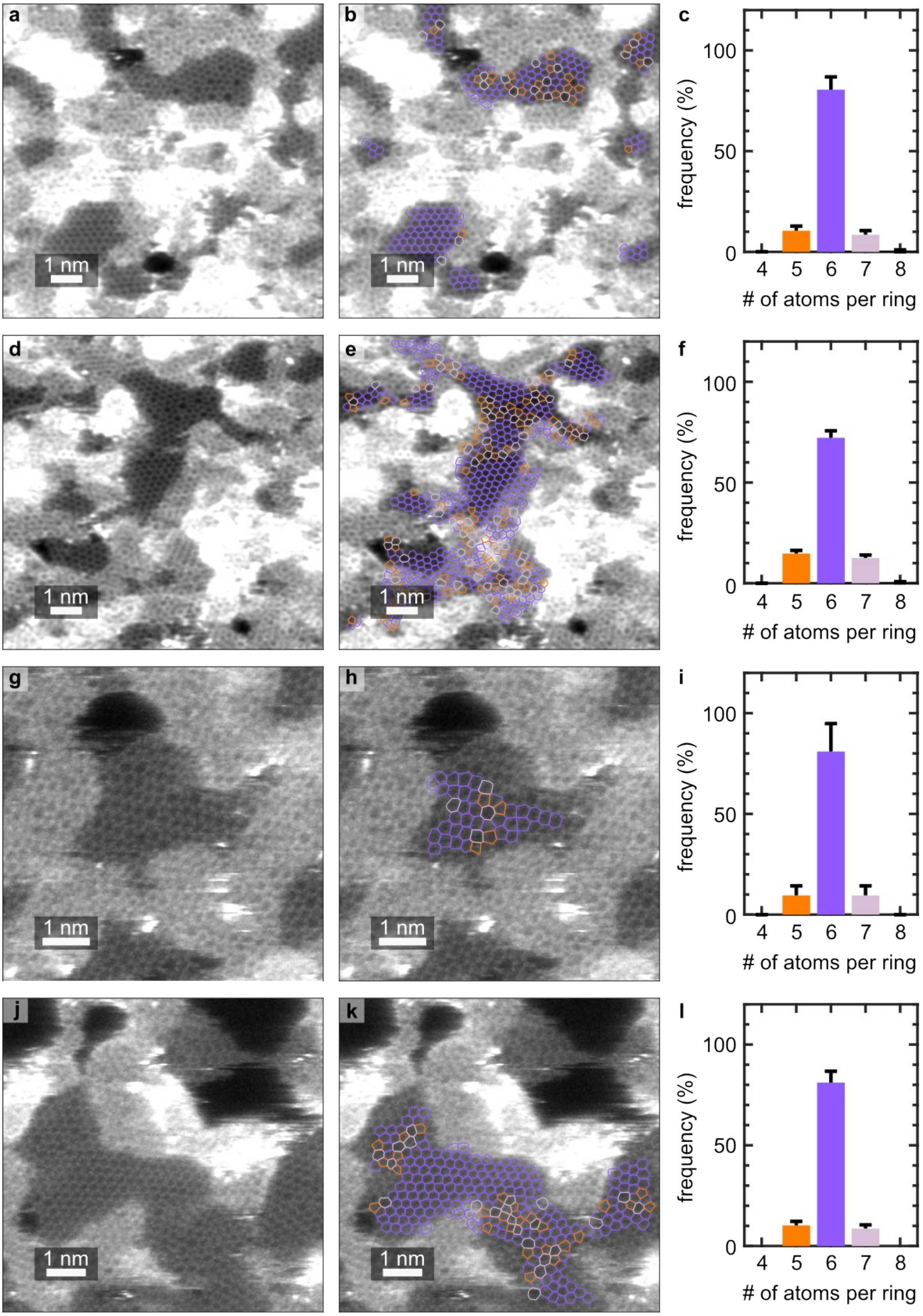

Figure S31: (**a**-**b**,**d**-**e**,**g**-**h**,**j**-**k**) Atomically resolved AC-STEM measurements of a defective film. Overlayed in panels (**b**,**e**,**h**,**k**) are the C-C bonds, 5-membered rings are dark orange, 7-membered are light purple and 6-membered are dark purple. Also shown in panels (**c**,**f**,**i**,**l**) are the histograms of the relative frequency of 4- to 8-membered rings.

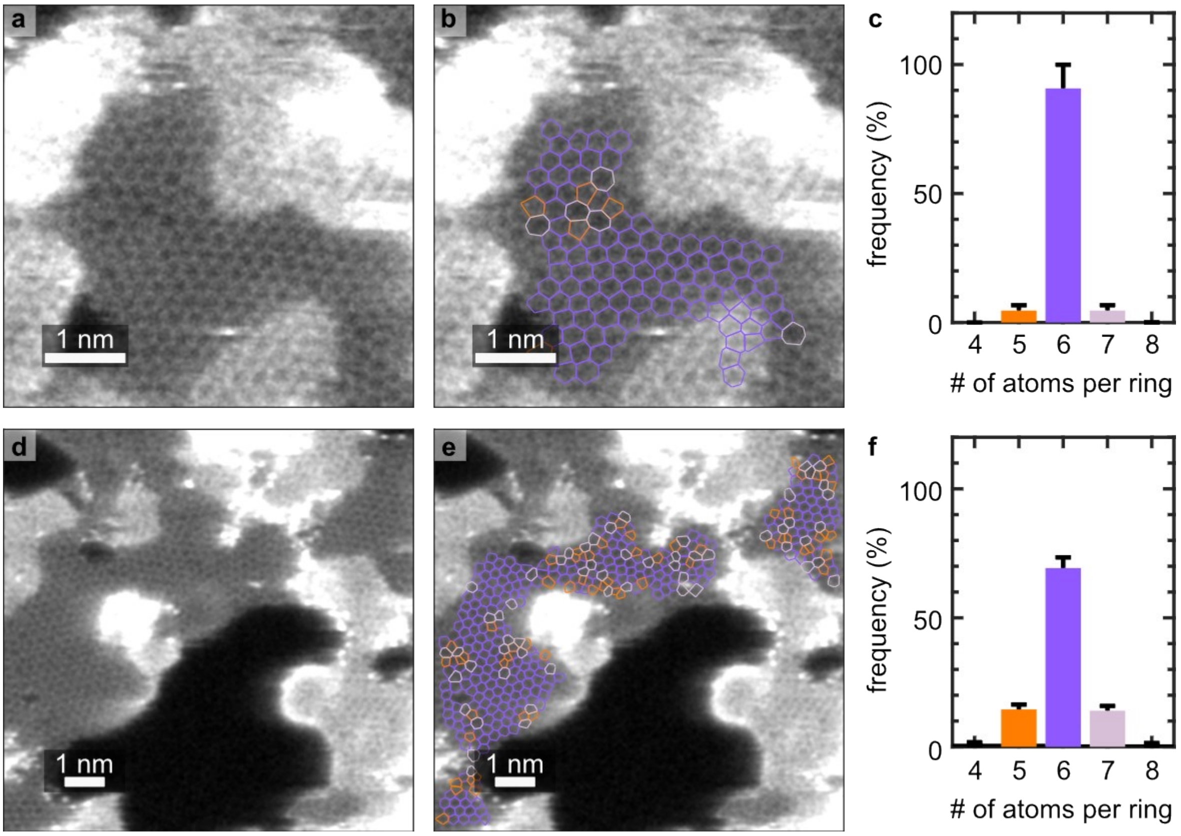

Figure S32: (**a-b**,**d-e**) Atomically resolved AC-STEM measurements of a defective film. Overlayed in panels (**b**,**e**) are the C-C bonds, 5-membered rings are dark orange, 7-membered are light purple and 6-membered are dark purple. Also shown in panels (**c**,**f**) are the histograms of the relative frequency of 4- to 8-membered rings.

Figure S33

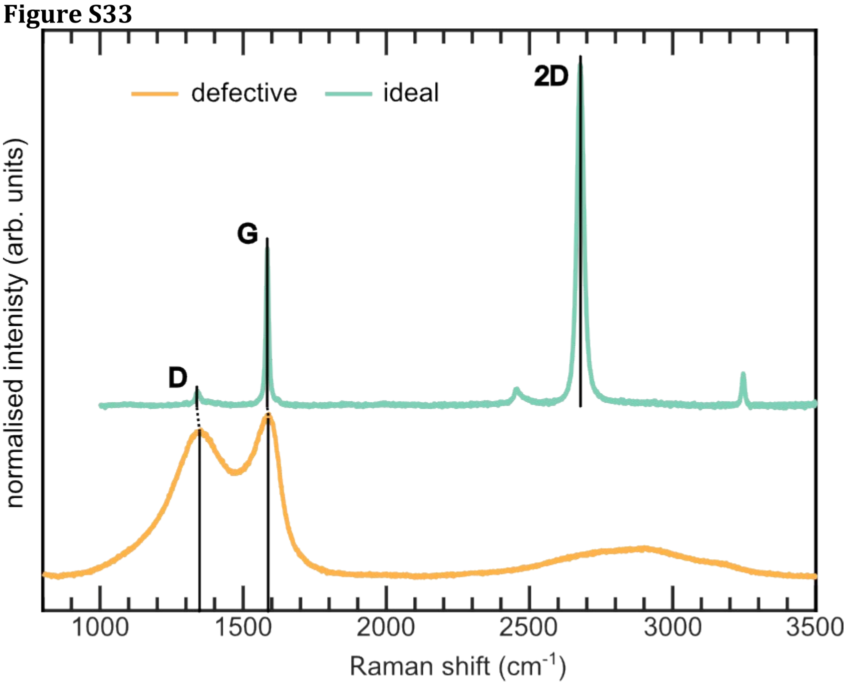

Figure S33: Raman spectra of an ideal graphene film (top, green) and a defective graphene film (bottom, orange) free-standing on a TEM grid. In each case, spectra were obtained from five random locations and subsequently averaged, baseline-corrected, normalised to the intensity of the G band and offset on the y-axis for ease of visual comparison. The D, G and 2D bands are indicated on the spectra. Determining the position of the 2D band in the Raman spectra of defective graphene films is challenging as the second order spectrum contains broad and strongly overlapping contributions from numerous overtones and combination modes, including the D+D'', 2D, D+D' and 2D' bands. These data indicate that the defective graphene film can be characterised as an early-to-mid "stage 2" carbon according to the amorphisation trajectory defined by Ferrari and Robertson (a graphitic layer with an increasing number of structural defects).[38]

References

1. B. P. Klein, M. A. Stoodley, M. Edmondson, L. A. Rochford, M. Walker, L. Sattler, S. M. Weber, G. Hilt, L. B. Williams and T.-L. Lee, *Applied Physics Letters*, 2022, **121**, 191603.
2. T.-L. Lee and D. A. Duncan, *Synchrotron Radiation News*, 2018, **31**, 16-22.
3. F. J. Giessibl, *Review of Scientific Instruments*, 2019, **90**, 011101.
4. F. Albrecht, F. Bischoff, W. Auwärter, J. V. Barth and J. Repp, *Nano Letters*, 2016, **16**, 7703-7709.
5. A. Preobrajenski, A. Generalov, G. Ohrwall, M. Tchaplyguine, H. Tarawneh, S. Appelfeller, E. Frampton and N. Walsh, *Journal of Synchrotron Radiation*, 2023, **30**, 831-840.
6. W. E. S. Unger, A. Lippitz, C. Wöll and W. Heckmann, *Fresenius' Journal of Analytical Chemistry*, 1997, **358**, 89-92.
7. C. J. Fisher, R. Ithin, R. G. Jones, G. J. Jackson, D. P. Woodruff and B. C. C. Cowie, *Journal of Physics: Condensed Matter*, 1998, **10**, L623.
8. F. Schreiber, K. A. Ritley, I. A. Vartanyants, H. Dosch, J. Zegenhagen and B. C. C. Cowie, *Surface Science*, 2001, **486**, L519-L523.
9. V. I. Nefedov, V. G. Yarzhemsky, I. S. Nefedova, M. B. Trzhaskovskaya and I. M. Band, *Journal of Electron Spectroscopy and Related Phenomena*, 2000, **107**, 123-130.
10. S. Doniach and M. Sunjic, *Journal of Physics C: Solid State Physics*, 1970, **3**, 285.
11. V. Blum, R. Gehrke, F. Hanke, P. Havu, V. Havu, X. Ren, K. Reuter and M. Scheffler, *Computer Physics Communications*, 2009, **180**, 2175-2196.
12. S. J. Clark, M. D. Segall, C. J. Pickard, P. J. Hasnip, M. I. J. Probert, K. Refson and M. C. Payne, 2005, **220**, 567-570.
13. J. P. Perdew, K. Burke and M. Ernzerhof, *Physical Review Letters*, 1996, **77**, 3865-3868.
14. V. G. Ruiz, W. Liu, E. Zojer, M. Scheffler and A. Tkatchenko, *Physical Review Letters*, 2012, **108**, 146103.
15. Y. Zhao and D. G. Truhlar, *Theoretical Chemistry Accounts*, 2008, **120**, 215-241.
16. M. A. Stoodley, L. A. Rochford, T.-L. Lee, B. P. Klein, D. A. Duncan and R. J. Maurer, *Physical Review Letters*, 2024, **132**, 196201.
17. S.-P. Gao, C. J. Pickard, A. Perlov and V. Milman, *Journal of Physics: Condensed Matter*, 2009, **21**, 104203.
18. L. Triguero, L. G. M. Pettersson and H. Ågren, *Physical Review B*, 1998, **58**, 8097-8110.
19. B. P. Klein, S. J. Hall and R. J. Maurer, *Journal of Physics: Condensed Matter*, 2021, **33**, 154005.
20. M. Schmid, H.-P. Steinrück and J. M. Gottfried, *Surface and Interface Analysis*, 2014, **46**, 505-511.
21. A. Hjorth Larsen, J. Jørgen Mortensen, J. Blomqvist, I. E. Castelli, R. Christensen, M. Dułak, J. Friis, M. N. Groves, B. Hammer, C. Hargus, E. D. Hermes, P. C. Jennings, P. Bjerre Jensen, J. Kermode, J. R. Kitchin, E. Leonhard Kolsbjerg, J. Kubal, K. Kaasbjerg, S. Lysgaard, J. Bergmann Maronsson, T. Maxson, T. Olsen, L. Pastewka, A. Peterson, C. Rostgaard, J. Schiøtz, O. Schütt, M. Strange, K. S. Thygesen, T. Vegge, L. Vilhelmsen, M. Walter, Z. Zeng and K. W. Jacobsen, *Journal of Physics: Condensed Matter*, 2017, **29**, 273002.
22. Y. Zhao and D. G. Truhlar, *Organic Letters*, 2006, **8**, 5753-5755.
23. D. J. Morgan, *Surface and Interface Analysis*, 2023, **55**, 567-571.
24. A. L. Walter, S. Nie, A. Bostwick, K. S. Kim, L. Moreschini, Y. J. Chang, D. Innocenti, K. Horn, K. F. McCarty and E. Rotenberg, *Physical Review B*, 2011, **84**, 195443.
25. A. J. Marsden, M.-C. Asensio, J. Avila, P. Dudin, A. Barinov, P. Moras, P. M. Sheverdyaeva, T. W. White, I. Maskery, G. Costantini, N. R. Wilson and G. R. Bell, *physica status solidi (RRL) – Rapid Research Letters*, 2013, **7**, 643-646.
26. B. P. Klein, A. Ihle, S. R. Kachel, L. Ruppenthal, S. J. Hall, L. Sattler, S. M. Weber, J. Herritsch, A. Jaegermann, D. Ebeling, R. J. Maurer, G. Hilt, R. Tonner-Zech, A. Schirmeisen and J. M. Gottfried, *ACS Nano*, 2022, **16**, 11979-11987.
27. C. F. P. Kastorp, D. A. Duncan, M. Scheffler, J. D. Thrower, A. L. Jørgensen, H. Hussain, T.-L. Lee, L. Hornekær and R. Balog, *Nanoscale*, 2020, **12**, 19776-19786.
28. J. Hermann and A. Tkatchenko, *Physical Review Letters*, 2020, **124**, 146401.
29. T. Mizoguchi, I. Tanaka, S.-P. Gao and C. J. Pickard, *Journal of Physics: Condensed Matter*, 2009, **21**, 104204.
30. A. Stukowski, *Modelling and Simulation in Materials Science and Engineering*, 2010, **18**, 015012.
31. D. P. Woodruff, *Reports on Progress in Physics*, 2005, **68**, 743.
32. M. J. Bedzyk and G. Materlik, *Physical Review B*, 1985, **32**, 6456-6463.
33. B. W. Batterman, *Physical Review*, 1964, **133**, A759-A764.
34. J. Zegenhagen and A. Kaimirov, *The X-ray Standing Wave Technique*, 2013.

35. S. T. Skowron, V. O. Koroteev, M. Baldoni, S. Lopatin, A. Zurutuza, A. Chuvilin and E. Besley, *Carbon*, 2016, **105**, 176-182.
36. J. Kotakoski, J. C. Meyer, S. Kurasch, D. Santos-Cottin, U. Kaiser and A. V. Krasheninnikov, *Physical Review B*, 2011, **83**, 245420.
37. J. C. Meyer, F. Eder, S. Kurasch, V. Skakalova, J. Kotakoski, H. J. Park, S. Roth, A. Chuvilin, S. Eyhusen, G. Benner, A. V. Krasheninnikov and U. Kaiser, *Physical Review Letters*, 2012, **108**, 196102.
38. A. C. Ferrari and J. Robertson, *Physical Review B*, 2000, **61**, 14095-14107.
